# Supplementary material for: Computational modelling reveals the influence of object similarity and proximity on visually guided movements
Source: PeerJ. 2025 Feb 25;13:e18953. doi: 10.7717/peerj.18953 (PMC11869896; doi:10.7717/peerj.18953)
Supplement: Supplemental Information 1 [file peerj-13-18953-s001.zip › CoRLEGO Simulation Code/All in one/documentation_v1_3.pdf]

COSIVINA: A Matlab Toolbox to Compose, Simulate, and Visualize Neurodynamic Architectures

Sebastian Schneegans
Institut für Neuroinformatik
Ruhr-Universität Bochum

Version 1.3, January 2015

Contents

1 Introduction 6
1.1 About this Document . . . 6
1.2 Overview of the Framework . . . 6
1.3 Object-Oriented Programming and Terminology . . . 7
1.4 Preparing the Framework for Use . . . 8

I Structure of the Framework 9

2 Element Class 9
2.1 Common Properties . . . 9
2.1.1 parameters . . . 9
2.1.2 components . . . 10
2.1.3 label . . . 10
2.2 Methods . . . 10
2.2.1 init . . . 10
2.2.2 step . . . 11
2.2.3 close . . . 11
2.2.4 copy . . . 11
2.2.5 addInput . . . 11
2.2.6 getParameterList . . . 12
2.3 Note on Size Parameters . . . 12

3 Simulator Class 12
3.1 Methods for Creating and Expanding the Simulator . . . 12
3.1.1 Constructor . . . 12
3.1.2 addElement . . . 13
3.1.3 addConnection . . . 13
3.1.4 copy . . . 14
3.2 Methods for Running the Simulator . . . 14
3.2.1 run . . . 14
3.2.2 init . . . 14
3.2.3 step . . . 15

3.2.4 close . . . 15
3.3 Element Access Methods . . . 15
3.3.1 isElement . . . 15
3.3.2 getElement . . . 15
3.3.3 getComponent . . . 15
3.3.4 setElementParameters . . . 15
3.4 Methods to Assist in Debugging and Optimization . . . 16
3.4.1 tryInit and tryStep . . . 16
3.4.2 stepWithTimer and runWithTimer . . . 16

4 GUI **16**
4.1 GUI Layout . . . 17
4.2 Parameter Panel . . . 18
4.3 Methods . . . 18
4.3.1 Constructor . . . 18
4.3.2 addVisualization . . . 19
4.3.3 addControl . . . 19
4.3.4 connect . . . 20
4.3.5 run . . . 20
4.3.6 init . . . 20
4.3.7 step . . . 20
4.3.8 updateVisualizations . . . 21
4.3.9 checkAndUpdateControls . . . 21

II Class Reference **22**

5 Elements **22**
5.1 Dynamic Elements . . . 22
5.1.1 NeuralField . . . 22
5.1.2 MemoryTrace . . . 22
5.1.3 DynamicVariable . . . 23
5.1.4 SingleNodeDynamics . . . 23
5.2 Interaction Kernels . . . 25
5.2.1 GaussKernel1D . . . 25
5.2.2 GaussKernel2D . . . 26
5.2.3 GaussKernel3D . . . 26
5.2.4 MexicanHatKernel1D . . . 27
5.2.5 MexicanHatKernel2D . . . 28
5.2.6 MexicanHatKernel3D . . . 28
5.2.7 LateralInteractions1D . . . 29
5.2.8 LateralInteractions2D . . . 30
5.2.9 LateralInteractions3D . . . 31
5.2.10 KernelFFT . . . 32
5.2.11 LateralInteractionsDiscrete . . . 33
5.2.12 WeightMatrix . . . 34
5.2.13 AdaptiveWeightMatrix . . . 34
5.3 Dimensional Reduction and Expansion . . . 35
5.3.1 SumDimension . . . 35
5.3.2 SumAllDimensions . . . 35

5.3.3 ExpandDimension . . . 36
5.3.4 ExpandDimension2D . . . 36
5.3.5 DiagonalSum . . . 37
5.3.6 DiagonalExpansion . . . 37
5.3.7 ScalarToGaussian . . . 37
5.4 Basic Mathematical Operations . . . 38
5.4.1 ScaleInput . . . 38
5.4.2 SumInputs . . . 38
5.4.3 ShiftInput . . . 39
5.4.4 PointwiseProduct . . . 39
5.4.5 Convolution . . . 40
5.4.6 Interpolation1D . . . 40
5.5 Output Functions . . . 41
5.5.1 Sigmoid . . . 41
5.5.2 HalfWaveRectification . . . 41
5.6 Static Stimuli . . . 41
5.6.1 BoostStimulus . . . 41
5.6.2 GaussStimulus1D . . . 42
5.6.3 GaussStimulus2D . . . 42
5.6.4 GaussStimulus3D . . . 43
5.6.5 CustomStimulus . . . 43
5.6.6 NormalNoise . . . 44
5.7 Time-Dependent Stimuli and Switches . . . 44
5.7.1 TimedSwitch . . . 44
5.7.2 TimedGaussStimulus1D . . . 44
5.7.3 TimedGaussStimulus2D . . . 45
5.7.4 TimedCustomStimulus . . . 46
5.7.5 CustomStimulusSequnce . . . 46
5.8 History . . . 46
5.8.1 History . . . 46
5.8.2 RunningHistory . . . 47
5.9 Image Acquisition and Processing . . . 47
5.9.1 CameraGrabber . . . 47
5.9.2 ImageLoader . . . 48
5.9.3 ColorExtraction . . . 48
5.10 Motor control . . . 49
5.10.1 AttractorDynamics . . . 49
5.10.2 DynamicRobotController . . . 49
5.10.3 MobileRobotArena . . . 50

6 Controls **51**
6.1 ParameterSlider . . . 51
6.2 ParameterSwitchButton . . . 52
6.3 ParameterDropdownSelector . . . 52
6.4 GlobalControlButton . . . 53
6.5 PresetSelector . . . 54
6.6 RobotArenaControlPanel . . . 54

7 Visualizations **55**
7.1 MultiPlot . . . 55
7.2 XYPlot . . . 56
7.3 SlicePlot . . . 57
7.4 ScaledImage . . . 58
7.5 RGBImage . . . 59
7.6 SurfacePlot . . . 59
7.7 KernelPlot . . . 59
7.8 StaticText . . . 60
7.9 TimeDisplay . . . 60

III Expanding the Framework **62**

8 Overview **62**

9 Creating New Element Classes **62**
9.1 General Structure . . . 62
9.2 Parameters . . . 63
9.3 Components . . . 64
9.4 Constructor . . . 64
9.5 Initialization . . . 65
9.6 Simulation Step . . . 65
9.7 Close External Connections . . . 65

10 Creating New Control Classes **66**
10.1 Constructor . . . 66
10.2 Connecting the Control to a Simulator . . . 67
10.3 Initialization . . . 67
10.4 Check Control . . . 67
10.5 Update Control . . . 67

11 Creating New Visualization Classes **67**
11.1 Contructor . . . 68
11.2 Connecting the Visualization to a Simulator . . . 68
11.3 Initialization . . . 69
11.4 Update Visualization . . . 69

IV Tutorials and Examples **70**

12 Using the Graphical User Interface **70**

13 Building a Simple DNF Architecture **72**

14 Running a Simulation in Offline Mode **75**

15 Creating a Graphical User Interface **77**
15.1 Creating the GUI Object 77
15.2 Adding Visualizations 78
15.3 Adding Controls to Change Element Parameters 80
15.4 Adding Global Controls 81

16 Using the GUI in Customized Simulations **82**

17 Building Architectures with Two-Dimensional Fields **86**
17.1 Fields, Inputs, and Lateral Interactions 86
17.2 Projections between Fields of Different Dimensionality 88

18 General Hints for Efficient DNF Architectures **89**
18.1 Connecting Fields of Different Dimensionality 89
18.2 Scaling and Re-using Operations 89
18.3 Order of Elements 89

1 Introduction

1.1 About this Document

The goal of this document is to enable the users of the COSIVINA toolbox to quickly build DNF architectures, design GUIs for them, and run their models in the way that is most appropriate for their requirements. This document does not provide a general introduction to Dynamic Neural Field Theory, its applications or the underlying mathematics (see http://www.robotics-school.org/ for introductory lectures and materials).

The document is structured into four major parts: The first describes the key components of the framework. The second one provides a reference of the available classes, specifically the elements that can be used to construct an architecture and the controls and visualizations for the design of a custom GUI. The third part describes how to expand the framework by adding new classes. The fourth part provides tutorials and examples for using this framework, including the construction of architectures and GUIs and different ways to run simulations for testing and tuning neurodynamic models. You may jump directly to this last part to get a quick idea of how to use this framework.

1.2 Overview of the Framework

The COSIVINA toolbox is intended as a tool to quickly create Dynamic Neural Field (DNF) architectures in Matlab and simulate their activation time course either in an interactive mode with a GUI, or in an offline mode. The software was developed at the Institut für Neuroinformatik at the Ruhr-Universität Bochum, where it is used both in teaching the concepts of DNFs and in research. It is published under the Simplified BSD License.

The basic idea for the framework is to divide a DNF architecture into individual *elements* that can be implemented as objects of different classes. Assume for example we have a simple one-dimensional dynamic neural field, which has lateral interactions (typically local excitiation and surround inhibition) and receives several inputs, often modeled as Gaussian functions. We can then divide this architecture up into the following elements: The dynamic neural field itself (the distribution of activation that changes according to a dynamic field equation); the lateral interaction kernel that is convolved with the field; and several Gaussian stimuli. These elements are connected to each other: The stimuli feed into the neural field, the element for the lateral interactions receives input from the field and feeds back to it. Larger architectures containing many neural fields that interact with each other through mutual projections or other operations can be segmented in the same way.

The dynamics of the architecture (the change of its state over time) is simulated using the Euler method: The rate of change for each dynamic element is determined for equidistant time steps and assumed fixed for the duration of the step. The segmentation into elements is done in such a way that the operation to be performed in each step can be computed for each element separately, based only on the element's own state, its direct *inputs* and the element's *parameters*.

In the framework, an architecture is constructed by adding elements and specifying their parameters and their connections to each other. The architecture can be run (i.e. the change of its state over time be simulated) either in individual steps or for a fixed duration. The states of of all element can then be accessed, analyzed or plotted, their parameters or states changed, and the simulation continued thereafter. Moreover, the framework offers tools to create graphical user interfaces (GUIs) that can be used to run the architecture while visualizing the states (like activation patterns or outputs) of some or all of its elements online. These GUIs allow online changes of parameters via control elements (sliders, buttons,

and edit fields). The settings of an architecture (its elements, their parameters and the connections between them) can be stored in a parameter file, and the architecture can be created again by loading from this file. The framework uses the JSON format (see www.json.org) to store parameter files, and utilizes the Open Source toolbox JSONlab for saving and loading these files.

As a typical usage of this framework for research in the field of behavioral/neural modeling, we envision something like the following: You start with an idea for a DNF model that performs a certain task. You implement that model in the DNF framework, e.g. by writing a script that creates the required elements and their connectivity, using the provided classes. Then you create a GUI for this architecture, which shows you the activations of all fields and maybe the results of some other operations that go on in each step of the simulation. Add some controls to change inputs to the system and key parameters, and try it out. You may have to do some debugging of the connectivity before the simulation actually runs; the framework provides some tools to assist you here. Then find out whether the architecture behaves the way you expected, at least qualitatively. You may have to change or add some elements or connections before it looks good. In the next step, to get the actual behavior that you want out of the system, tuning of the parameters will almost certainly be necessary. The standard GUI allows you to access all parameters of the elements in your architecture, so that you can change their behavior while the simulation is running. You may also add more controls to have a more direct way to change some key properties. Store the parameter settings that are promising in parameter files as you work on the model. At some point you may want to start running standardized trials with the model, perhaps with some fixed sequence of inputs. You can then move to running the model in an offline mode (without the GUI, though perhaps still with some visualization or analysis as the simulation runs). Just load the architecture from the parameter files and write a script that sets up the desired time course and the necessary analysis of the results. And from here, you can switch between GUI and the offline mode to test the model, analyze its behavior, improve and expand it.

1.3 Object-Oriented Programming and Terminology

Working with the DNF framework does not require detailed knowledge of object-oriented programming, and providing such knowledge would be beyond the scope of this document (see the Matlab help for this). We will, however, give a very brief overview of the concepts and terminology of object-oriented programming in Matlab to facilitate understanding of our framework. The central idea of object-oriented programming is to structure the program code (or parts of it) into *classes*, which combine data structures and functions that act on them. A class in Matlab can be written as a single m-file that contains a class definition, which specifies the name of the class and its relationship to other classes. This class definition is typically followed by a list of persistent variables of this class, called *properties*. A class can furthermore define functions, which typically act on these properties. These functions are called *methods* of the class.

To use the class and its methods in scripts or other functions, it is typically necessary to instantiate it – that is, to create an *object* of that class. This can be done by calling a *constructor* function for the class, which has the same name as the class itself. The constructor call returns a concrete variable, which contains all the properties of the class (analogous to the fields in a struct). One can access these properties and call the methods of the class via the dot-notation (in the form `objectName.propertyName` or `objectName.methodName(...)`). Some properties may not be accessible (or not accessible for writing) except via methods of the class. It is possible and common to instantiate mul-

tiple objects of the same class. These objects then have the same structure and offer the same methods, but the content of their internal variables may be entirely different.

The DNF framework strongly relies on so called *handle classes*: When an object of a handle class is created, it is instantiated in memory and a handle to it is returned as variable in the workspace. This handle allows access to the class's properties and methods via the dot-notation in the same way as in non-handle classes. However, when copying this variable, only the handle to the existing object is copied, not the object itself. This is similar to graphics handles in Matlab, which can be copied without multiplying the graphics object. The handle class object itself is destroyed when all handles referring to it are removed from the workspace.

We will furthermore use some fixed terms below that are specific to our framework (and not to be confused with general object-oriented programming terminology). In particular, we will refer to the *parameters* and *components* of an element. These two terms refer to different types of properties of the element classes, that are distinguished by their role for the behavior of the element, not by their implementation.

1.4 Preparing the Framework for Use

To use the framework, download the compressed Matlab sources and unpack them in a folder of your choice. Then add the subfolders `base`, `controls`, `elements`, `examples` (optional), `mathTools`, and `visualizations` to your Matlab path. You can do so manually via the entry `Set Path ...` in the Matlab `File` menu (choose `Add with subfolders`, then save the settings for future Matlab sessions), or call the function `setpath` in the COSIVINA base directory.

The full functionality of COSIVINA is supported by Matlab R2011a and later. The framework can also be used with earlier versions (back to at least R2009a), but then requires a small adjustment to run: In the file `base/Element.m`, replace the class definition

```
classdef Element < matlab.mixin.Copyable
```

with

```
classdef Element < handle
```

(both forms are prepared in the file, just comment/uncomment the appropriate line). With this change, the `copy` functions of the `Element` and `Simulator` classes are no longer functional. Except for this, the framework remains fully functional, including the creation and use of GUIs for interactive simulations.

In order to save and load architecture settings to/from configuration files, you additionally need the Open Source toolbox JSONlab. You can obtain it from

`http://sourceforge.net/projects/iso2mesh/files/jsonlab/`

The current version of COSIVINA has been tested with JSONlab versions 0.9.1 to 1.0. The location of this toolbox also needs be added to the Matlab path. The `setpath` function will do so if a folder `jsonlab` exists either as a subfolder in the COSIVINA base folder or on the same level as the COSIVINA base folder itself.

To test the toolbox, call one of the scripts from the `examples` folder. For instance, call `launcherOneLayerField.m` from the Matlab command line (the `launcher...` files create a DNF architecture and accompanying GUI and then run that GUI). This should open a GUI window with a running DNF model, in which you can change field parameters and input settings via sliders. Press the `Quit` button to close the simulation. You can also run the example scripts, which are explained in detail in the last part of this document.

Part I
Structure of the Framework

2 Element Class

Different types of elements (like neural fields, lateral interactions, and projections between fields) are implemented as different classes in the DNF framework. These classes are all derived from the abstract superclass `Element`, which defines a common structure for all elements.

2.1 Common Properties

2.1.1 parameters

When we talk about the *parameters* of a class, we refer to a certain subset of its properties (class variables) which together fully define the behavior of an element. Each element class contains as one (constant) property a struct named `parameters`. This struct contains the names of all parameters of the element together with information about whether they may be changed during a simulation. It is important to note that this struct does not contain the parameter values themselves. Instead, it only provides the information which parameters the element has and how they behave, which is important e.g. to automatically generate GUI panels that allow manipulation of parameters online. The parameter values themselves are stored in individual properties of the element class.

For instance, for the class `NeuralField`, the `parameters` struct has the following form:

```
>> NeuralField.parameters
ans =
    size: 0
     tau: 1
       h: 1
    beta: 1
```

This tells us that the elements of this class have four parameters, namely `size`, `tau`, `h` and `beta`. The integers following the parameter names inform us whether the parameter may be changed online (see 9 for details). If we create an object of this class, we get a list of its properties:

```
>> nf = NeuralField
ans =
  NeuralField handle
  Properties:
              parameters: [1x1 struct]
              components: {'input'  'activation'  'output'  'h'}
    defaultOutputComponent: 'output'
                    size: [1 1]
                     tau: 10
                       h: -5
                    beta: 4
                   input: []
              activation: []
```

```
                output: []
                 label: ''
               nInputs: 0
         inputElements: {}
       inputComponents: {}
```

We see that the four parameters listed above now appear as properties of the object, among others. Their values are the actual parameter values (e.g. `h = -5`), in this case the default values assigned to them by the class constructor.

2.1.2 components

A second subset of properties is called *components*. These vectors or matrices contain the state of the element during the simulation (for example, the current activation distribution of a neural field) or the results of the computation performed by an element in every simulation step (for example, the convolution of an input with an interaction kernel). These components can serve as inputs for other elements or be plotted in a GUI's visualizations. Analogously to the parameters, there is a single constant property `components` in each class. We can view it without actually creating an object of the class:

```
>> NeuralField.components
ans =
  'input'  'activation'  'output'  'h'
```

This property lists all of the class's components as a cell of strings.

As shown in this listing, the `NeuralField` class has the components `input`, `activation`, `output`, and `h`. (Note that a single property can serve as both a parameter and a component, as is the case here for the resting level `h`.) The components are typically created as empty matrices in the element constructor and then brought to their correct size during the initialization (see below).

In addition to the `component` property, each element furthermore has a property `defaultOutputComponent`. This string gives the name of the property that is used as the default when projecting from that element to other elements. For most elements, including the `NeuralField` class, the name of this component is simply `output`:

```
>> NeuralField.defaultOutputComponent
ans =
  output
```

2.1.3 label

Each element that is added to the architecture in a `Simulator` object must have a unique label. A valid label can be any non-empty character string. The label is used to refer to this element in the simulator, e.g. when specifying the connectivity between elements or accessing an element for analysis or parameter changes.

2.2 Methods

2.2.1 init

The `init` method initializes the element. After initialization, all components of the elements are created as vectors or matrices of the correct size (filled either with zeros or with appropriate values), so that they can serve as valid inputs for other elements. The `init` method

furthermore performs all preparatory computations and may prepare internal data structures for the `step` method to be executed. For certain elements, it also opens a connection to hardware or interfaces to other programs.

2.2.2 step

The method `step(time, deltaT)` performs one simulation step (Euler step) at a simulation time given by the first argument and of a duration given by the second argument. It fetches input from other elements and updates the element's components as applicable. Note: The arguments `time` and `deltaT` are in practice only used by a small subset of element classes, and ignored in the step functions of other classes.

2.2.3 close

The `close` method disconnects the element from hardware or external interfaces. In pure simulation settings, use of this method is generally not necessary. In these cases, calling this method has no effect.

2.2.4 copy

The `copy` method creates a shallow copy of an element. The method is inherited from `matlab.mixin.Copyable`, the superclass of `Element`. Since all element classes are handle classes, a simple assignment does not copy the element, but only creates a new handle to an existing element. Consider the following example:

```
h1 = NeuralField('field u', 100, 10, -5);
h2 = h1;
h1.tau = 20;
h2.tau
ans =
    20
```

Here, the handles `h1` and `h2` refer to the same underlying element. When a parameter is changed via handle `h1`, the change will also appear when the element is accessed via handle `h2`. In contrast, the following code creates an actual copy of the element:

```
h1 = NeuralField('field u', 100, 10, -5);
h2 = h1.copy();
h1.tau = 20;
h2.tau
ans =
    10
```

The two handles now point to different elements, so a parameter change via one handle will not change the element accessed by the other handle.

2.2.5 addInput

The method `addInput(inputHandle, inputComponent, optArg)` is used to manually add an input to an element. The connections in an architecture are in practice stored in such a way that each element has a list of elements that it receives inputs from, and a list of the components of these elements that serve as inputs. When creating an architecture, the

connections between the elements are typically defined via the `addElement` function of the `Simulator` class (which calls this method internally), such that direct calls of the `addInput` method are not necessary in most cases.

2.2.6 getParameterList

This method returns a list of the element's parameter names as a cell array of strings.

2.3 Note on Size Parameters

The framework supports DNFs and connecting elements of any dimensionality. The activation states and outputs are realized in Matlab as matrices with a corresponding number of dimensions. Most elements have a parameter `size` that indicates the size of the field or other structures represented by the element. These size parameters are vectors with at least two elements. Following Matlab conventions, the first entry is interpreted as the number of rows of a matrix, and parameters referring to this first dimension are typically marked with the letter `y`. The second entry reflects the number of columns, and corresponding parameters are marked with the letter `x`. Parameters referring to the third dimension (if present) are marked with the letter `z` (this leads to the counter-intuitive sequence `y`, `x`, `z`). Higher dimensions are not assigned a specific letter.

One-dimensional data structures are by default stored as row vectors, and if a scalar is given as a size parameter, it is interpreted as the number of columns in a row vector (so a value `n` given as a size argument is interpreted as `[1, n]`). You can overwrite this default and employ column vectors by explicitly specifying a size as `[n, 1]`, but typically there should be no need for this.

3 Simulator Class

The `Simulator` class holds all elements of an architecture, manages the connections between them, allows to run the whole architecture as a coupled dynamic system and provides easy access to individual components. It furthermore contains methods to save an architecture to or load it from a configuration file. Below we describe the functions for creating, expanding, and using a `Simulator` object.

3.1 Methods for Creating and Expanding the Simulator

3.1.1 Constructor

A new `Simulator` object can be created by calling the constructor without any arguments, e.g. `sim = Simulator()`. Optional arguments can be provided in the form of parameter name / value pairs (where the parameter name is given as a character string). The parameters `'tZero'` (the starting time of the simulator, default is 0) and `'deltaT'` (the time step, default is 1) can be set, or an architecture can be loaded from a configuration file in JSON format by specifying the parameter `'file'` and the filename, or it can be loaded from a parameter structure by giving the parameter `'struct'` and the struct.

Examples

`sim = Simulator();` creates an empty simulator object with default settings

`sim = Simulator('deltaT', 0.1, 'tZero', 500);` creates an empty simulator object with a time step of 0.1 and a start time of 500

`sim = Simulator('file', 'threeLayerArchitecture.json');` creates a simulator object by loading the architecture and parameter settings from the specified JSON-file

`sim = Simulator('struct', threeLayerArchtecture);` creates a simulator object by loading the architecture and parameters from the struct `threeLayerArchtecture`; the struct must be a valid parameter struct, created e.g. by loading from a parameter file using the JSONlab toolbox

3.1.2 addElement

This method provides the key capacity of the `Simulator` class for constructing architectures, by a adding a new element to the `Simulator` object. The function is called in the form

```
sim.addElement(elementHandle, inputLabels, inputComponents,
  targetLabels, componentsForTargets)
```

The element handle is typically created by calling the constructor of the element that is to be added and providing the necessary parameters for the element. The four following arguments are all optional, and describe the connectivity of the new element to the existing elements of the architecture. They can each be either a single string or a cell array of strings, or be an empty matrix if no connections are to be specified.

- `inputLabels` - the labels of those existing elements in the architecture from which the newly added element receives inputs
- `inputComponents` - the components of the elements specified in `inputLabels` that are to be used as inputs for the new element; if the new element receives multiple inputs, both `inputLabels` and `inputComponents` should be cell arrays of strings, with each pair of entries from the two cells specifying one input; the whole argument `inputComponent` or individual elements of the cell array may be replaced by an empty array `[]`, in which case the default output component of the specified input elements is used
- `targetLabels` - the labels of those elements in the architecture that are to receive input from the new element
- `componentsForTarget` - the components of the new element that should serve as inputs for the elements specified in `targetLabels`; analogous to the `inputLabels` and `inputComponents` arguments, multiple targets may be specified through cell arrays of strings, and `componentsForTarget` may be omitted if the default output component of the new element is to be used

3.1.3 addConnection

This method creates one or more new connections between existing elements in the `Simulator` object. Typically, this method is not needed for building architectures, since connections can be created together with new elements using the method `addElement`; but the option to create new connections separately is provided for convenience. The method call takes the form

```
sim.addConnection(inputLabels, inputComponents, targetLabel)
```

with the following arguments:

- `inputLabels` - the labels of those existing elements in the architecture that are the source of the newly added connections; may be either a single string, or a cell array of strings
- `inputComponents` - the components of the elements specified in `inputLabels` that are to be used as input to the element specified by `targetLabel`; may be either a single string, or a cell array of strings (with the same size as `inputLabels`); if an empty array is provided for one or more entries in the cell array, the standard output component of the specified element is used as input
- `targetLabel` - the label of the existing element in the architecture that is the target of the new connections; must be a single string

3.1.4 copy

Creates a copy of the simulator object and all of its elements. Since the class `Simulator` is a handle class, a simple assignment does not copy the object, but only creates a new handle to the existing object (see 2.2.4 for detailed explanation). The copy method can be used to create different branches of a simulation, which can be run independently to directly compare how a model that is in a certain state behaves under different conditions.

For instance, you may have an architecture with a complex working memory representation, that takes some time to be built. You may then, after forming this representation, create several copies of the simulator object to test how the model behaves when different new inputs are applied. This way, you do not need to form the working memory representation repeatedly, and can be sure that you always start with the exact same state when comparing different conditions.

3.2 Methods for Running the Simulator

3.2.1 run

The method runs the simulator (i.e. performs simulation steps) until a specified time step is reached. It can initialize the simulator and close it after finishing if requested. It is called as `sim.run(tMax, initialize, close)`, where the boolean arguments `intialize` and `close` are both optional.

If the simulator is not yet initialized or if an initialization is explicitly requested by setting the argument `initialize` to `true`, the `init` method is called (see below), which initializes all elements and sets the time to `tZero`. Otherwise, the simulation continues from the current state of the simulator. This means that a single continuous run may be replaced by multiple successive calls of `run` (with increasing values for `tMax`), allowing e.g. to perform analysis of the simulator's state or change settings between the individual runs.

If the argument `close` is supplied and its value is `true`, all elements are closed after completion of the run by calling their `close` method (see `close` method below).

3.2.2 init

The `init` method initializes all elements in the simulator object (by calling each element's `init` method), and sets the simulator time property `t` to the value `tZero`. It takes no arguments and returns the initialized simulator object. If the simulator was already initialized, all information about its previous state is lost.

3.2.3 step

The `step` method performs a single simulation step (updating of the dynamical system's state according to the Euler method) by calling each element's `step` function, and increments the simulator's time property `t` by `deltaT`. It takes no arguments and returns the initialized simulator object.

Note: The elements are processed (i.e. their `step` functions called) in the order they were added to the simulator. The order may make a difference for the exact behavior of the system.

3.2.4 close

The `close` method terminates all external connections by calling each element's `close` method. Note: The `close` method is used primarily to disconnect elements that provide interfaces to hardware. It does not affect most other elements, and is typically not required in pure simulation settings.

3.3 Element Access Methods

3.3.1 isElement

The method call `sim.isElement(s)` checks whether the string `s` is the label of any element in the object `sim`, and returns a boolean value.

3.3.2 getElement

The method call `sim.getElement(s)` returns a handle to the element with label `s` if such an element exists in the simulator object, and an empty matrix otherwise.

3.3.3 getComponent

The method call `sim.getComponent(s, c)` with string arguments `s` and `c` returns the component with name `c` of element with label `s` if such exists. Otherwise it returns an empty matrix and throws a warning.

3.3.4 setElementParameters

The method call `setElementParameters(elementLabels, parameterNames, newValues)` allows the user to change parameter values of one or more elements in the simulator. The arguments `elementLabels` and `parameterNames` may be either strings or cell arrays of strings. You may either specify a single element and one or more parameters, or pairs of element labels and parameter names that each specify one parameter to be changed. The argument `newValues` must be a single value or a cell array of values, matching the number of specified parameters. The type of each entry in `newValues` must be appropriate for the corresponding parameter.

After setting the parameter values, the function will automatically re-initialize the affected elements and call the `step` function if this is necessary for the change to take effect (see Section 9 for details). This re-initialization may be required if a parameter value is not used in each step of an element, but only in some preparatory computation (like computing of an interaction kernel, which is done only once). The dynamic elements in the

framework (`NeuralField`, `MemoryTrace`, and `DynamicVariable`) do not have any parameters that would require re-initialization, so the state of the system is not altered by the `setElementParameters` functions.

Certain parameters are considered fixed and cannot be changed by this function. It is still possible to access and change these parameters manually, but it may require special care to do this, especially during a running simulation (for instance, changing the size of one element will often require adjustment of other elements as well). Using the `setElementParameters` is generally the safer and easier way to perform parameter changes.

3.4 Methods to Assist in Debugging and Optimization

3.4.1 tryInit and tryStep

The methods `tryInit` and `tryStep` perform the same operations as `init` and `step`, but provide additional information if an error occurs. In that case, the label of the element that caused the error is displayed together with all its properties. The `tryStep` function also displays information about the element's inputs, since a frequent cause of erros is mismatch between the sizes of connected elements. If no errors occur, the methods performs a valid initialization or step of the simulator object.

3.4.2 stepWithTimer and runWithTimer

The method `stepWithTimer` performs a simulation step while measuring the runtime of each element, and returns a vector of times with one entry for each element (ordered in the same way as the `Simulator` object's `elementLabels` property). The method `runWithTimer` can be used in the same way as `run`, but internally calls `stepWithTimer` and returns a vector of total runtimes for each element. An easy way to display the runtimes associated with each element is the following:

```
runtimes = sim.runWithTimer(100);
[sim.elementLabels', num2cell(runtimes)]
```

4 GUI

The graphical user interface of the framework makes it possible to change parameter values while a simulation is running, so that the behavior of an architecture can be observed and adjusted online. Currently there is one type of GUI implemented in the `StandardGUI` class. Each `StandardGUI` object is connected to a single `Simulator` object. A specific GUI is built up by arranging *visualizations* and *controls*: The visualizations are graphical elements like plots or images that show selected components of the architecture elements. Controls can be buttons, sliders, dropdown menus and other objects that are connected to selected parameters of the architecture elements. Certain controls can globally affect the simulation that is run in the GUI (e.g. pausing or ending the simulation). As a default interface to access and change any (non-fixed) parameters, the `StandardGUI` offers a parameter panel, implemented in the separate class `ParameterPanel`. In this panel, an element from the architecture can be selected via a dropdown menu. All of its parameters are then displayed and their values can be changed via edit fields.

The `StandardGUI` class can be used in two different ways: Either in a full online mode, in which the GUI itself controls the running simulation; or in a passive mode for visualization of externally controlled simulation only. The first mode is started by the method

`run`, the second mode is used by calling the methods `init`, `updateVisualizations`, and `checkAndUpdateControls`.

4.1 GUI Layout

The `StandardGUI` has one main figure window on which controls and visualizations can be arranged freely. To make the arrangement of items more convenient, two spatial grids over this window can be defined: The *visualizations grid* and the *controls grid*. When a new `StandardGUI` object is created, the positions and sizes of these grids can be specified. The grid position in the main window must be given in relative coordinates (as four-element vector, as used e.g. to place plots in Matlab). The grid size is given by a two-element vector, determining the number of cells vertically and horizontally. An example is shown in Figure 4.1. The locations of controls and visualizations can then be given as positions in the respective grid, and a size within the grid can be specified if they should span multiple grid cells. Alternatively, all positions can also specified manually as relative positions in the figure.

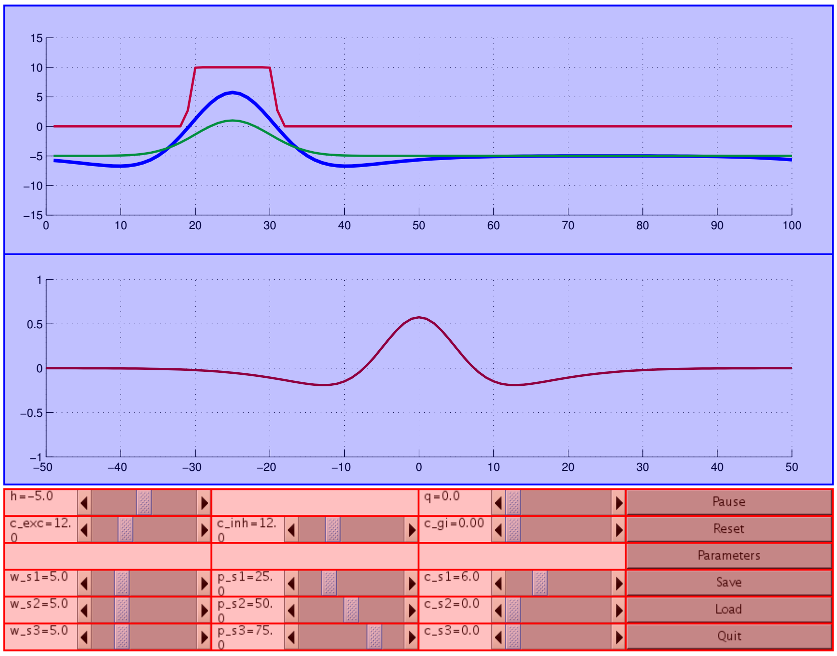

Figure 1: Layout of the GUI in `launcherField1l.m` using visualizations and controls grids. The 2×1 visualizations grid (blue) and the 6×4 controls grid (red) are overlaid over the actual GUI.

A position within the grid is described by a two-element vector `[v, h]` (vertical and horizontal position), with `[1, 1]` being the top left cell of the grid. While the typical usage is to describe positions by integer values in this grid, non-integer real values and values that fall outside of the specified grid are permissible.

For the visualizations grid, there is an additional parameter `visGridPadding` that determines a space left between the actual visualization and the border of the grid cell. It is given relative to the size of the figure window.

Note that the grids for controls and visualizations may also overlap. An example can be seen in the file `launcherCoupling.m`, where the controls grid covers the whole window (but no controls are actually placed in the area occupied by visualizations).

4.2 Parameter Panel

The parameter panel can be opened in a separate figure window via an appropriate button in the main window. By default, the parameter panel generates a list of all elements in the connected `Simulator` object and shows them in a dropdown menu. For the selected elements, it shows all of its parameters with an edit field containing the parameter values. The values may be changed for non-fixed element parameters, with the changes taking effect after the `Apply` button at the bottom of the panel is clicked. (Note: Changes made for one element are lost if the selection in the dropdown menu is changed without clicking `Apply` first.)

The element list for the parameter panel can be customized by specifying a list of entries and a list of the corresponding elements or element groups in the `StandardGUI` constructor. This customization supports three functions:

- to list the elements in the parameter panel with more descriptive labels than are actually used in the architecture;
- to eliminate elements from the parameter panel's dropdown menu, e.g. to make the list more concise by removing entries that do not have any changeable parameters or that should not be changed by the user;
- to group elements together, such that changes made via the parameter panel affect a group of elements rather than a single element.

The customization is performed via two parameters `elementGroups` and `elementsInGroups`. The first is a cell array of strings, with each string specifying one entry in the parameter panel's dropdown menu (you may use descriptive labels here, the entries do not have to match the actual element labels). The second parameter `elementsInGroups` specifies one or more elements (via their labels) that are to be associated with the corresponding entry in `elementGroups`. It is either a cell array of strings (if each menu entry is associated with only one element) or a cell array of cell arrays of strings (you may also mix string and cell array of string entries).

4.3 Methods

4.3.1 Constructor

A new `StandardGUI` object is created by calling the class constructor in the form

```
StandardGUI(simulatorHandle, figurePosition, pauseDuration, ...
      visGridPosition, visGridSize, visGridPadding, ...
      controlGridPosition, controlGridSize, ...
      elementGroups, elementsInGroups, figureTitle)
```

All arguments except `figurePosition` are optional.

- `simulatorHandle` - handle to the `Simulator` object that is to be run by the GUI; if the argument is 0, the GUI will be created without being connected to a specific simulator; it must then be connected later using the `connect` method or by specifying a simulator handle when calling `run`

- `figurePosition` - position of the GUI's main figure window as a four element vector `[posX, posY, width, height]`
- `pauseDuration` - duration of pause for every simulation step (default = 0.1, should be set lower for computationally costly simulations)
- `visGridPosition` - position of the visualizations grid in the GUI window in the format `[posX, posY, width, height]`, in normalized coordinates (relative to figure size)
- `visGridSize` - grid size of the visualizations grid as a two-element vector `[rows, cols]`
- `visGridPadding` - padding of the visualizations within the grid cells (relative to figure size), as scalar or as vector `[padHor, padVert]`
- `controlGridPosition` - position and size of the controls grid in the main figure window in relative coordinates (four element vector)
- `controlGridSize` - grid size of the controls grid (two-element vector)
- `elementGroups` - dropdown menu entries for the parameter panel (cell array of strings)
- `elementsInGroups` - labels of elements associated with dropdown menu entries (cell array of strings or cell array of cell array of strings)
- `figureTitle` - character string that is displayed in the GUI window's title bar

4.3.2 addVisualization

The method call

```
gui.addVisualization(visualization, positionInGrid, sizeInGrid, gridSelection)
```

adds the visualization specified in the first argument to the GUI. If the second argument is specified (as a two-element vector `[row, col]`), the visualization will be placed at this position in the GUI's visualization grid. If the third argument is specified (also a two-element vector), the visualization will span the specified number of grid cells vertically towards the bottom and horizontally to the right; otherwise, it will cover one grid cell. Alternatively, the position of the visualization can be specified directly in the visualization object, without making use of the GUI's visualizations grid. Finally, if you set the optional argument `gridSelection` to `'control'`, the visualization will be placed at the specified location in the control grid rather than the visualizations grid.

4.3.3 addControl

The method call

```
gui.addControl(control, positionInGrid, sizeInGrid, gridSelection)
```

adds the control specified in the first argument to the GUI. Position information can be specified analogously to the `addVisualization` method. Set the last parameter to `'visualization'` to place a control element in the visualizations grid.

4.3.4 connect

The method call

```
gui.connect(simulatorHandle)
```

sets the simulator object that is connected with the GUI (that is, which is run in the GUI). The method can be used if no simulator was specified in the constructor call for the GUI, or if the GUI should be connected to a different simulator than originally specified. An error is thrown if the new simulator does not contain the elements, parameters, and components that are specified in the GUI's controls and visualizations.

4.3.5 run

The method call

```
gui.run(tMax, initializeSimulation, closeSimulation, simulatorHandle)
```

runs the simulation in the GUI until it reaches simulation time `tMax`. If not specified, `tMax` is set to infinity by default, meaning that the simulation will run until stopped manually from the GUI. The optional boolean argument `intializeSimulation` specifies whether the connected `Simulator` object should be initialized upon start of the GUI. By default, the `Simulator` object is only initialized if it was not initialized before. If the `Simulator` object is already initialized, it will continue at its current state. The optional boolean argument `closeSimulation` specifies whether the `close` function of the simulator should be called when the GUI is closed (to close connections to external software or hardware; false by default, not needed in pure simulation settings). The optional argument `simulatorHandle` can be used to specify the simulator object that will be run in the GUI. This can be used if no simulator was specified when creating the GUI object, or when the GUI should be run with a different simulator object than originally specified (e.g. a new copy of the original simulator).

Note: If you quit the GUI via a GUI control, or if the GUI stops after reaching a specified simulation time `tMax`, the simulator object remains in the state that it last had in the GUI. If you call the `run` method again afterwards without explicitly requesting an initialization, you can continue the simulation from this point.

4.3.6 init

The method call `gui.init()` initializes the GUI and opens the GUI's main figure window. It should be used if the GUI is to be used in an offline mode, with visualizations updated by explicit function calls (see below). If you use the `run` function, the GUI is initialized automatically.

4.3.7 step

The method call `gui.step()` performs a single step in the connected simulator object, unless the simulation is paused. It then updates all visualizations, checks the controls for changes and applies those to the simulator object, and updates the controls themselves where necessary. The method also checks for changes in the flags that can be set by the global control buttons (like pause, save, and quit), and performs the corresponding operations (like starting a file selection dialog or closing the GUI).

4.3.8 updateVisualizations

The method call `gui.updateVisualizations()` updates all visualizations in the GUI to reflect the current state of the connected simulator object.

4.3.9 checkAndUpdateControls

The method call `checkAndUpdateControls` checks all controls for changes and applies these to simulator object. It also updates the state of control elements to reflect parameter changes (e.g. slider positions). NOTE: Control buttons (like pause, save, and quit) will set the appropriate flags in the StandardGUI object, but have no further effects. The effects have to be implemented manually in the code calling this method.

Part II
Class Reference

5 Elements

5.1 Dynamic Elements

5.1.1 NeuralField

Description A dynamic neural field of any size and dimensionality, or a set of dynamic nodes. In each step, the field activation $u(x)$ is updated according to the Amari equation, using the sum of all inputs $I(x)$, and the field output $f(u(x))$ is computed from the activation via a sigmoid function:

$$\tau \dot{u}(x) = -u(x) + h + I(x) \tag{1}$$

$$f(u(x)) = \frac{1}{1 + \exp(\beta u(x))} \tag{2}$$

Lateral interactions in the field have to be implemented by separate connective elements that receive input from the field and project back to it.

Parameters

- `size` - size of the field (determines the size of the three components listed below)
- `tau` - time constant of the field
- `h` - field resting level
- `beta` - steepness parameter of the sigmoid output function

Components

- `activation` - field activation
- `output` - field output (sigmoid of the activation)

(Note: The component `input` that existed for this element in version 1.0 has been removed to improve performance.)

Inputs The `NeuralField` may have an arbitrary number of inputs, which have to be either scalar or have the same size as the field.

5.1.2 MemoryTrace

Description A memory trace for a dynamic neural field of any size and dimensionality, or for a set of dynamic nodes. The memory trace is typically operated with a significantly higher time constant (i.e. slower dynamics) than a field. It typically receives the output of a neural field as input I. If the input at any point exceeds a specified threshold θ, the memory trace is updated according to the rule

$$\dot{m}(x) = \begin{cases} \frac{1}{\tau_{build}}(-m(x) + I(x)), & \text{if } I(x) > \theta \\ \frac{1}{\tau_{decay}}(-m(x)), & \text{else} \end{cases} \tag{3}$$

If the input is below θ everywhere, the memory trace does not change.

Parameters

- `size` - size of the memory trace
- `tauBuild` - time constant for the accumulation of the memory trace (default = 100)
- `tauDecay` - time constant for the decay of the memory trace (default = 1000)
- `threshold` - threshold applied to the input to determine whether and where a memory trace should accumulate (default = 0.5)

Components

- `output` - memory trace
- `activeRegions` - regions in the current input that exceed the threshold (binary vector/matrix of the same size as the input)

Inputs The `MemoryTrace` must have exactly one input whose size matches the element's `size` parameter.

Note: If you use the output of a field as input for a memory trace, you should use the default threshold value of 0.5 (this corresponds to an activation value of 0 in the field).

5.1.3 DynamicVariable

Description A matrix of dynamic variables. The summed input is interpreted as a rate of change, which is scaled with a time constant to change the state of the dynamic variables. The dynamic variables behave like a field or set of dynamic nodes without a resting level, that is in the absence of external input they simply maintain their state.

Parameters

- `size` - size of the matrix of dynamic variables
- `tau` - time constant
- `initialState` - state of the dynamic variables at initialization

Components

- `state` - current state of the dynamic variables
- `initialState` - state of the dynamic variables at initialization

Inputs The `DynamicVariable` may have an arbitrary number of inputs, which have to be either scalar or match the element's `size` parameter.

5.1.4 SingleNodeDynamics

Note: This class is intended for easy visualization and analysis of a node's dynamic behavior (in particular for creating plots of rate of change vs. possible activation value, showing attractors and repellors). If you want only the basic behavior of a discrete dynamic node, use the `NeuralField` class instead!

Description The class creates a single dynamic node with a sigmoid (logistic) output function, tunable self-excitation and Gaussian noise:

$$\tau \dot{u} = -u + h + c_{exc} f(u) + I + q\xi \tag{4}$$

Here, τ is a time constant, u is the node activation, h its resting level, c_{exc} the strength of self-excitation (may be negative to create self-inhibition), f is the sigmoid output function, I is the sum of external inputs, q is the noise level and ξ random variable following a normal distribution.

In addition, the class computes the rate of change $\dot{u}$ – given the current external input – for a range of possible activation values u of the node, and determines approximate attractor and repellor states within this range (zero crossings of the rate of change). The components provided by this class can be used in particular for plotting the node dynamics using the `XYPlot` visualization (see `launcherTwoNeuronSimulator` for an example of this).

Parameters

- `tau` - time constant of the node dynamics
- `h` - resting level of node activation
- `beta` - steepness of sigmoid (logistic) output function
- `selfExcitation` - strength of self-excitation
- `noiseLevel` - strength of random Gaussian noise in node activation
- `range` - two-element vector specifying the range of activation values for which the rate of change is computed
- `resolution` - resolution (or actually, step size) with which the given range is sampled for computation of rate of change values

Components

- `input` - sum of all external inputs to the node
- `activation` - current activation value
- `output` - current output of the node (sigmoid of the activation)
- `h` - resting level of node activation
- `rateOfChange` - rate of change for the node's activation in the current step
- `samplingPoints` - vector of activation values (specified by parameters `range` and `resolution`) for which the rate of change is computed
- `sampledRatesOfChange` - vector of rates of change, computed at each sampling point (given the current external input to the node)
- `attractorStates` - vector of approximate activation values within `range` at which attractor states exist (vector may be empty and change size as parameters or inputs change)

- `attractorRatesOfChange` - vector of rates of change at the attractor states (same size as `attractorStates`, all values should be close to zero, provided for plotting attractors)
- `repellorStates` - vector of approximate activation values within `range` at which repellor states exist (vector may be empty and change size as parameters or inputs change)
- `repellorRatesOfChange` - vector of rates of change at the repellor states (same size as `repellorStates`, all values should be close to zero, provided for plotting attractors)

Inputs The `SingleNodeDynamics` can have an arbitrary number of scalar inputs.

5.2 Interaction Kernels

5.2.1 GaussKernel1D

Description Convolves an input with a Gaussian interaction kernel along one dimension (horizontally). Typically this element is used for one-dimensional inputs, but it may also be used with two-dimensional inputs if convolution only along the horizontal dimension is desired (otherwise use GaussKernel2D).

Parameters

- `size` - size of the input and output of the element
- `sigma` - width parameter of the Gaussian kernel
- `amplitude` - amplitude of the Gaussian kernel
- `circular` - flag indicating whether the convolution is performed in a circular fashion (i.e., values at the left end of the input can affect the output at the right end and vice versa); default value is true
- `normalized` - flag indicating whether the Gaussian kernel is normalized before scaling it with the amplitude (for a normalized kernel, the amplitude equals the integral over the kernel; without normalization, the amplitude equals the value of the kernel at the center); default value is true
- `cutoffFactor` - sets the cutoff factor for the kernel (to make computation faster, the kernel function is cut off at a certain multiple of sigma, ignoring those parts of the kernel where interactions strengths are very small); advised range is 3 to 5 (5 being the default); set to `inf` to always use full kernel

Components

- `output` - result of the input's convolution with the interaction kernel
- `kernel` - interaction kernel (incorporating the `amplitude` parameter)

Inputs The `GaussKernel1D` element must have exactly one input whose size matches the element's `size` parameter.

5.2.2 GaussKernel2D

Description Convolves a two-dimensional input with a two-dimensional Gaussian interaction kernel. The convolution is internally performed separately along the two dimensions for computational efficiency.

Parameters

- `size` - size of the input and output of the element
- `sigmaY` - width parameter of the Gaussian kernel for the vertical axis
- `sigmaX` - width parameter of the Gaussian kernel for the horizontal axis
- `amplitude` - amplitude of the Gaussian kernel
- `circularY` - flag indicating whether the vertical convolution is performed in a circular fashion (i.e., values at the left end of the input can affect the output at the right end and vice versa); default value is true
- `circularX` - flag indicating whether the horizontal convolution is performed in a circular fashion; default value is true
- `normalized` - flag indicating whether the Gaussian kernel is normalized before scaling it with the amplitude; default value is true
- `cutoffFactor` - sets the cutoff factor for the kernel (see `GaussKernel1D`); default value is 5

Components

- `output` - the result of the input's convolution with the interaction kernel
- `kernelX` - the horizontal component of the interaction kernel (incorporating the `amplitude` parameter)
- `kernelY` - the vertical component of the interaction kernel

Inputs The `GaussKernel2D` element must have exactly one input whose size matches the element's `size` parameter.

5.2.3 GaussKernel3D

Description Connective element that performs a convolution with a Gaussian kernel over three-dimensional space. The convolution is internally performed separately along each dimension for computational efficiency.

Parameters

- `size` - size of the input and output of the element (must be a three-element vector)
- `sigmaY`, `sigmaX`, `sigmaZ` - width parameters of the Gaussian kernel for each dimension
- `amplitude` - amplitude of the Gaussian kernel

- `circularY`, `circularX`, `circularZ` - binary flags indicating whether the convolution is performed in a circular fashion along each dimension (i.e., values at the left end of the input can affect the output at the right end and vice versa); default value is true
- `normalized` - flag indicating whether the Gaussian kernel is normalized before scaling it with the amplitude; default value is true
- `cutoffFactor` - sets the cutoff factor for the kernel (see `GaussKernel1D`); default value is 5

Components

- `output` - the result of the input's convolution with the interaction kernel
- `kernelY`, `kernelX`, `kernelZ` - one-dimensional components of the interaction kernel

Inputs The `GaussKernel3D` element must have exactly one input whose size matches the element's `size` parameter.

5.2.4 MexicanHatKernel1D

Description Convolves the input with a difference of two Gaussian interaction kernels along one dimension (horizontally). The element can be used in particular to implement an interaction pattern of local excitation and surround inhibition ("mexican hat"). Typically this element is used for one-dimensional inputs, but it may also be used with two-dimensional inputs if convolution only along the horizontal dimension is desired.

Parameters

- `size` - size of the input and output of the element
- `sigmaExc` - width parameter of the excitatory kernel component
- `amplitudeExc` - amplitude of the excitatory kernel component
- `sigmaInh` - width parameter of the inhibitory kernel component
- `amplitudeInh` - amplitude of the inhibitory kernel component (the inhibitory component is subtracted from the excitatory one, so a positive value of `amplitudeInh` corresponds to actual inhibition)
- `circular` - flag indicating whether the convolution is performed in a circular fashion; default value is true
- `normalized` - flag indicating whether each kernel component is normalized before scaling it with the amplitude; default value is true
- `cutoffFactor` - sets the cutoff factor for the kernel (see `GaussKernel1D`); default value is 5

Components

- `output` - result of the input's convolution with the interaction kernel
- `kernel` - interaction kernel

Inputs The `MexicanHatKernel1D` must have exactly one input whose size matches the element's `size` parameter.

5.2.5 MexicanHatKernel2D

Description Convolves the input with a difference of two Gaussian interaction kernels along two dimensions. The element can be used in particular to implement an interaction pattern of local excitation and surround inhibition ("mexican hat").

Parameters

- `size` - size of the input and output of the element
- `sigmaExcY, sigmaExcX` - width parameters of the excitatory kernel component along the vertical and horizontal axes
- `amplitudeExc` - amplitude of the excitatory kernel component
- `sigmaInhY, sigmaInhX` - width parameters of the inhibitory kernel component along the vertical and horizontal axes
- `amplitudeInh` - amplitude of the inhibitory kernel component (the inhibitory component is subtracted from the excitatory one, so a positive value of `amplitudeInh` corresponds to actual inhibition)
- `circularY, circularX` - flags indicating whether the convolution is performed in a circular fashion along each dimension; default value is true
- `normalized` - flag indicating whether each kernel component is normalized before scaling it with the amplitude; default value is true
- `cutoffFactor` - sets the cutoff factor for the kernel (see `GaussKernel1D`); default value is 5

Components

- `output` - result of the input's convolution with the interaction kernel
- `kernelExcX` - horizontal excitatory component of the interaction kernel (scaled with excitatory amplitude)
- `kernelExcY` - vertical excitatory component of the interaction kernel (not scaled)
- `kernelInhX` - horizontal inhibitory component of the interaction kernel (scaled with inhibitory amplitude)
- `kernelInhY` - vertical inhibitory component of the interaction kernel (not scaled)

Inputs The `MexicanHatKernel2D` must have exactly one input whose size matches the element's `size` parameter.

5.2.6 MexicanHatKernel3D

Description Convolves the input with a difference of two Gaussian interaction kernels along three dimensions. The element can be used in particular to implement an interaction pattern of local excitation and surround inhibition ("mexican hat").

Parameters

- `size` - size of the input and output of the element (must be a three-element vector)
- `sigmaExcY`, `sigmaExcX`, `sigmaExcZ` - width parameters of the excitatory kernel component for each dimension
- `amplitudeExc` - amplitude of the excitatory kernel component
- `sigmaInhY`, `sigmaInhX`, `sigmaInhZ` - width parameters of the inhibitory kernel component for each dimension
- `amplitudeInh` - amplitude of the inhibitory kernel component
- `circularY`, `circularX`, `circularZ` - binary flags indicating whether the convolution is performed in a circular fashion along each dimension (i.e., values at the left end of the input can affect the output at the right end and vice versa); default value is true
- `normalized` - flag indicating whether the Gaussian kernel is normalized before scaling it with the amplitude; default value is true
- `cutoffFactor` - sets the cutoff factor for the kernel (see `GaussKernel1D`); default value is 5

Components

- `output` - the result of the input's convolution with the interaction kernel
- `kernelExcY`, `kernelExcX`, `kernelExcZ` - one-dimensional excitatory components of the interaction kernel
- `kernelInhY`, `kernelInhX`, `kernelInhZ` - one-dimensional inhibitory components of the interaction kernel

Inputs The `MexicanHatKernel3D` element must have exactly one input, a three-dimensional matrix whose size matches the element's `size` parameter.

5.2.7 LateralInteractions1D

Description Combines a Mexican-hat style convolution kernel with a global interaction component (in which the sum of the input is computed, scaled with a constant factor and globally added to the output). This class offers a compact form to add the full lateral interactions typically used in DNFs to a model. If no global interactions are needed, `MexicanHatKernel1D` should be used instead.

Parameters

- `size` - size of the input and output of the element
- `sigmaExc` - width parameter of the excitatory kernel component
- `amplitudeExc` - amplitude of the excitatory kernel component
- `sigmaInh` - width parameter of the inhibitory kernel component

- `amplitudeInh` - amplitude of the inhibitory kernel component (the inhibitory component is subtracted from the excitatory one, so a positive value of `amplitudeInh` corresponds to actual inhibition)
- `amplitudeGlobal` - amplitude of the global kernel component (a negative value must be given to create global inhibition)
- `circular` - flag indicating whether the convolution is performed in a circular fashion; default value is true
- `normalized` - flag indicating whether each local kernel component is normalized before scaling it with the amplitude
- `cutoffFactor` - sets the cutoff factor for the kernel (see `GaussKernel1D`); default value is 5

Components

- `output` - result of the input's convolution with the interaction kernel
- `kernel` - local interaction kernel (sum of scaled excitatory and inhibitory components)
- `amplitudeGlobal` - global interaction weight (scalar value)
- `fullSum` - sum of the input over the horizontal dimension

Inputs The `LateralInteractions1D` element must have exactly one input whose size matches the element's `size` parameter.

5.2.8 LateralInteractions2D

Description Connective element that performs a 2D convolution with a Mexican hat kernel (difference of two Gaussians) with a global component. The element also provides the sum of the element's input (typically the output of a neural field) along the horizontal, vertical, and both dimensions, to be used for projections onto lower-dimensional structures. If no global interactions are needed, `MexicanHatKernel2D` should be used instead.

Parameters

- `size` - size of the input and output of the element
- `sigmaExcY, sigmaExcX` - width parameters of the excitatory kernel component along the vertical and horizontal axes
- `amplitudeExc` - amplitude of the excitatory kernel component
- `sigmaInhY, sigmaInhX` - width parameters of the inhibitory kernel component along the vertical and horzontal axes
- `amplitudeInh` - amplitude of the inhibitory kernel component (the inhibitory component is subtracted from the excitatory one, so a positive value of `amplitudeInh` corresponds to actual inhibition)
- `amplitudeGlobal` - amplitude of the global kernel component (a negative value must be given to create global inhibition)

- `circularY, circularX` - flags indicating whether the convolution is performed in a circular fashion along each dimension; default value is true
- `normalized` - flag indicating whether each local kernel component is normalized before scaling it with the amplitude
- `cutoffFactor` - sets the cutoff factor for the kernel (see `GaussKernel1D`); default value is 5

Components

- `output` - result of the input's convolution with the interaction kernel
- `kernelExcX` - horizontal excitatory component of the interaction kernel (scaled with excitatory amplitude)
- `kernelExcY` - vertical excitatory component of the interaction kernel (not scaled)
- `kernelInhX` - horizontal inhibitory component of the interaction kernel (scaled with inhibitory amplitude)
- `kernelInhY` - vertical inhibitory component of the interaction kernel (not scaled)
- `amplitudeGlobal` - global interaction weight (scalar value)
- `horizontalSum` - sum of the input over the horizontal dimension
- `verticalSum` - sum of the input over the vertical dimension
- `fullSum` - sum of the input over both horizontal and vertical dimension

Inputs The `LateralInteractions2D` element must have exactly one input whose size matches the element's `size` parameter.

5.2.9 LateralInteractions3D

Description Connective element that performs a 3D convolution with a Mexican hat kernel (difference of two Gaussians) with a global component.

Parameters

- `size` - size of the input and output of the element (must be a three-element vector)
- `sigmaExcY`, `sigmaExcX`, `sigmaExcZ` - width parameters of the excitatory kernel component for each dimension
- `amplitudeExc` - amplitude of the excitatory kernel component
- `sigmaInhY`, `sigmaInhX`, `sigmaInhZ` - width parameters of the inhibitory kernel component for each dimension
- `amplitudeInh` - amplitude of the inhibitory kernel component
- `amplitudeGlobal` - amplitude of the global kernel component (a negative value must be given to create global inhibition)

- `circularY`, `circularX`, `circularZ` - binary flags indicating whether the convolution is performed in a circular fashion along each dimension (i.e., values at the left end of the input can affect the output at the right end and vice versa); default value is true
- `normalized` - flag indicating whether the Gaussian kernel is normalized before scaling it with the amplitude; default value is true
- `cutoffFactor` - sets the cutoff factor for the kernel (see `GaussKernel1D`); default value is 5

Components

- `output` - the result of the input's convolution with the interaction kernel
- `kernelExcY`, `kernelExcX`, `kernelExcZ` - one-dimensional excitatory components of the interaction kernel
- `kernelInhY`, `kernelInhX`, `kernelInhZ` - one-dimensional inhibitory components of the interaction kernel
- `fullSum` - sum of the input over all three dimensions

Inputs The `LateralInteractions3D` element must have exactly one input whose size matches the element's `size` parameter.

5.2.10 KernelFFT

Description Connective element that performs an n-dimensional convolution with a Mexican hat kernel (difference of two Gaussians) with a global component, using the FFT method (the kernel and the input are transformed into Fourier space, multiplied, and the result is transformed back). This is often faster than the direct convolution method implemented in elements like GaussKernel1D or LateralInteractions2D.

This element can be used for all convolutions with Gaussian kernels or difference-of-Gaussian kernels (with or without global component). The computational cost is the same for all of these, and depends only on the size of the input, not the width of the kernel. It is therefore especially suited if the kernel is large (high sigma value) relative to the size of the input. The input may be of any dimensionality.

By default, the convolution in the FFT method is always performed in a circular fashion along all dimensions. The element allows an emulation of a non-circular convolution by automatically padding the input with zeros and then cutting off the borders from the result. Note that this can become extremely slow for higher-dimensional inputs; elements with direct convolution should be used in these cases.

Parameters

- `size` - size of input and output of the convolution (an n-element vector for n-dimensional input)
- `sigmaExc` - an n-dimensional vector specifying the width of the excitatory Gaussian component in the n dimensions
- `amplitudeExc` - a scalar value specifying the strength of the excitatory Gaussian component

- `sigmaInh` (optional) - an n-dimensional vector specifying the width of the inhibitory Gaussian component in the n dimensions
- `amplitudeInh` (optional) - a scalar value specifying the strength of the inhibitory Gaussian component (larger positive value creates stronger inhibition; default is zero)
- `amplitudeGlobal` (optional) - a global (constant) component of the interaction kernel; set to negative value to obtain global inhibition (default is zero)
- `circular` (optional) - and n-dimensional vector of binary values, specifying for each dimension whether the convolution should be performed in a circular fashion (default is true for all dimensions, non-circular convolution can be very slow for large inputs)
- `normalized` (optional) - a binary value specifying whether the Gaussian kernels are normalized (so their integral over space is one) before scaling with the amplitudes (default is true)
- `paddingFactor` (optional) - scalar value determining how much padding is appended to the input for non-circular convolution (as a multiple of the larger sigma value along each dimension); larger values produce more complete removal of circular effects, but make convolution slower (default value is 5)

Components

- `output` - the result of the input's convolution with the interaction kernel

Inputs The `KernelFFT` element must have exactly one input whose size matches the element's `size` parameter.

5.2.11 LateralInteractionsDiscrete

Description Element to mediate lateral interactions within a set of discrete dynamic nodes. It comprises purely local interactions (typically self-excitation), computed by scaling each entry in the input vector with the parameter `amplitudeExc`, and purely global interactions (typically global inhibition), computed by scaling the sum over all input entries with the parameter `amplitudeGlobal`.

Parameters

- `size` - size of the input and output of the element
- `amplitudeExc` - amplitude of the local interactions
- `amplitudeGlobal` - amplitude of the global interactions (a negative value must be given to create global inhibition)

Components

- `output` - result of the weighting operation
- `amplitudeExc` - amplitude of local interactions
- `amplitudeGlobal` - amplitude of global interactions

Inputs The `LateralInteractionsDiscrete` element must have exactly one input whose size matches the element's `size` parameter.

5.2.12 WeightMatrix

Description Connective element that computes its output O by multiplying its input I (a row vector) with a weight matrix:

$$O = I \cdot W \tag{5}$$

The weight matrix should be specified in the constructor call and determines the size of the expected input and the produced output. For and input of size $[1, N]$ and an desired output of size $[1, M]$, the weight matrix must have the size $[N, M]$.

Parameters

- `size` - size of the output
- `weights` - weight matrix

Components

- `output` - result of the matrix multiplication (a row vector)

Inputs The `WeightMatrix` element must have exactly one input, a row vector whose length matches the number of rows in the weight matrix.

5.2.13 AdaptiveWeightMatrix

Description Connective element that multiplies its first input with a weight matrix W, and updates the weight matrix according to a Hebbian learning rule. The output O is computed as

$$O = I_1 \cdot W, \tag{6}$$

where both the input I_1 and the output O are row vectors.

The weight matrix is updated at each position according to the rule

$$\Delta W(i,j) = \text{learningRate} \cdot G \cdot [I_2(j) - W(i,j)] \cdot I_1(i) \tag{7}$$

Here, G is an optional third input (scalar) that gates the learning. I_2 is the same size as O and should typically be derived from the same structure (such as a neural field) that also receives input from the `AdaptiveWeightMatrix`. W is initialized as a zero matrix.

Parameters

- `size` - size of the weight matrix (input size $\times$ output size)
- `learningRate` - learning rate for weight update

Components

- `output` - result of the matrix multiplication (a row vector)
- `weights` - weight matrix

Inputs The `AdaptiveWeightMatrix` element can have either two or three inputs. The first two inputs I_1 and I_2 are processed as described above. The optional third input G should be a scalar value that scales the weight update (it may be a boolean to create a strict gating of the weight update).

5.3 Dimensional Reduction and Expansion

5.3.1 SumDimension

Description Element that computes the sum over one or more dimensions of its input. Optionally, the result can be scaled with a fixed amplitude, and the order of dimensions in the formed sum can be changed.

Parameters

- `sumDimensions` - dimension(s) of the input over which the sum is computed (either a single integer value or a vector of integers)
- `size` - size of the element's output
- `amplitude` (optional) - scalar value that is multiplied with the formed sum (default value is 1)
- `dimensionOrder` (optional) - order in which the non-singleton dimensions of the formed sum should appear in the output; must be a vector containing the integers `[1, ..., nDim]` exactly once, where nDim is the number of dimensions specified in `outputSize`; this argument has no effect if the output is a vector (the argument `outputSize` can then be used to determine whether the output is a row or column vector); default is `[1, ..., nDim]`)

Components

- `output` - result of the summation and scaling; if the result of the summing operation is a one-dimensional vector, its shape (row or column vector) is controlled by the parameter `size`.

Inputs The `SumDimension` element must have exactly one input. After summing along the specified dimensions, its size must match the element's `size` parameter.

5.3.2 SumAllDimensions

Description Computes separate sums for the horizontal and vertical dimension as well as the full sum (over both dimensions) for a two-dimensional input.

Parameters

- `size` - size of the element's input (from which the output sizes are derived)

Components

- `horizontalSum` - sum of the input over the horizontal (second) dimension, transposed to yield a row vector (with size $[1, n]$ for an input of size $[n, m]$)
- `verticalSum` - sum of the input over the vertical (second) dimension (with size $[1, m]$ for an input of size $[n, m]$)
- `fullSum` - sum of the input over both dimensions (yielding a scalar value)

Inputs The `SumAllDimensions` element must have exactly one input whose size matches the element's `size` parameter.

5.3.3 ExpandDimension

Description Expands a lower-dimensional input into a higher-dimensional matrix. Corresponding dimensions in input and output can be specified freely.

Parameters

- `inputSize` - size of the input matrix
- `outputSize` - size of the output matrix
- `dimensionMapping` - vector of integers specifying which dimension in the input matrix should be mapped to which dimension in the output matrix; the size of this vector must match the number of dimensions in the input matrix; The i-th entry determines to which dimension in the output the i-th dimension of the input is mapped; if the input is a vector, this argument may be either a single integer, or a two-element vector (since vectors are treated as two-dimensional matrcies in Matlab); singleton dimensions in the input may be mapped to arbitrary dimensions in the output

Components

- `output` - result of the expansion of the input

Inputs The `ExpandDimension` element must have exactly one input whose size matches the parameter `inputSize`.

5.3.4 ExpandDimension2D

Description Expands a one-dimensional input along a specified axis to form a two-dimensional array.

Parameters

- `expandDimension` - dimension along which the input is to be expanded (1 for vertical, 2 for horizontal; the input is always transposed in such a way that it runs along the dimension that is expanded)
- `size` - size of the element's output (after expansion)

Components

- `output` - result of the expansion of the input

Inputs The `ExpandDimension2D` element must have exactly one input. After expanding along the specified dimension, its size must match the element's `size` parameter. The orientation of the input is not relevant.

5.3.5 DiagonalSum

Description Computes sum of a square array along the second diagonal.

Parameters

- `inputSize` - size of the two-dimensional input (can be either a scalar value or a two-element vector with equal entries)
- `amplitude` - scalar value that is multiplied with the formed sum

Components

- `output` - the scaled diagonal sum of the input as a row vector (for an input array of size $[n, n]$, the size of the output is $[1, 2n - 1]$)

Inputs The `DiagonalSum` element must have exactly one input, a square two-dimensional array whose size matches the element's `size` parameter.

5.3.6 DiagonalExpansion

Description Expands a one dimensional input diagonally into a square matrix. The input must have an odd number of columns, such that the center of the input is expanded along the main diagonal of the resulting square matrix.

Parameters

- `inputSize` - size of the one-dimensional input (must be row vector and the number of columns must be odd)
- `amplitude` - scalar value that is multiplied with the input before expansion

Components

- `output` - the scaled square matrix resulting from the expansion (for an input vector of size $[1, 2n - 1]$, the output has size $[n, n]$)

5.3.7 ScalarToGaussian

Description Creates a one-dimensional Gaussian output pattern centered on a scalar input value. The input value can be scaled and an offset added to map the value range of the scalar to the desired range in the output (the full output has the range `[1, size(2)]`). Note: The difference to the element `GaussStimulus1D` is that `ScalarToGaussian` receives an input and is updated in every step, whereas in `GaussStimulus1D` the position of the Gaussian is determined by a parameter and the output is only updated if that parameter is changed.

Parameters

- `size` - size of the output
- `inputScale` - scale for the input value; the center of the Gaussian is determined as $position = inputScale \cdot input + inputOffset$
- `inputOffset` - constant offset for the input value, see above
- `sigma` - width parameter of the Gaussian
- `amplitude` - strength of the Gaussian
- `circular` - flag indicating whether the output is defined in a circular fashion (i.e., if the Gaussian is centered near the left end of the output, it can flow over into the right end and vice versa); default value is true
- `normalized` - flag indicating whether the Gaussian output is normalized before scaling it with the amplitude (for a normalized Gaussian, the integral is one if the amplitude is one; without normalization, the value at the center is one if the amplitude is one); default value is false

Components

- `output` - the Gaussian pattern
- `position` - the center of the Gaussian (the scaled input with offset added)

Inputs The `ScalarToGaussian` element takes exactly one input, a scalar value that determines the center of the Gaussian.

5.4 Basic Mathematical Operations

5.4.1 ScaleInput

Description Scales an input with a constant factor.

Parameters

- `size` - size of the input and output of the element
- `amplitude` - scalar value with which the input is scaled

Components

- `output` - result of the scaling (input multiplied with `amplitude` parameter)

Inputs The `ScaleInput` element must have exactly one input whose size matches the element's `size` parameter.

5.4.2 SumInputs

Description Computes the sum of multiple inputs.

Parameters

- `size` - size non-scalar inputs and output of the element

Components

- `output` - sum of all inputs

Inputs The `SumInputs` element may have an arbitrary number of inputs, which have to be either scalar or whose size has to match the element's `size` parameter.

5.4.3 ShiftInput

Description Shifts an input array by a specified value along the horizontal and/or vertical axis.

Parameters

- `size` - size of the input and output of the element
- `shiftValue` - a two-element integer vector specifying the value by which the input is shifted along the first (vertical) and second (horizontal) dimension
- `amplitude` - scalar value that is multiplied with the shifted input
- `circular` - flag indicating whether the shift should be performed circularly
- `fillValue` - value with which those parts of the output array are filled that are not occupied by the shifted input (for non-circular shifts)

Components

- `output` - the result of shift and scaling operations

Inputs The `ShiftInput` element must have exactly one input whose size matches the element's `size` parameter.

5.4.4 PointwiseProduct

Description Performs a pointwise multiplication between two inputs of equal size (with arbitrary dimensionality).

Parameters

- `size` - size of the two inputs and the output

Components

- `output` - pointwise product of the inputs

Inputs The `PointwiseProduct` element takes exactly two inputs, whose size must match the element's `size` parameter.

5.4.5 Convolution

Description Performs a convolution between two one- or two-dimensional inputs. Either input can be flipped horizontally and vertically (i.e. rotated by 180°) to effectively perform a correlation.

Parameters

- `size` - size of the result of the convolution operation
- `flipInputs` - integer value coding which of the inputs should be flipped; in the constructor, two separate boolean arguments are required to indicate separately whether the first and/or the second input should be flipped before the convolution
- `shape` - shape of the convolution, as in the Matlab function `conv2`; can be 'full', 'same', or 'valid'

Components

- `output` - the result of the convolution

Inputs The `Convolution` element takes exactly two inputs, and the size of the matrix resulting from the convolution between them must match the `size` parameter of the element.

5.4.6 Interpolation1D

Description Computes the output by interpolating from the input at specified positions, using the Matlab function `interp1`.

Parameters

- `size` - size of the output of the element
- `interpolationPoints` - vector containing the positions at which the input vector should be interpolated
- `method` - interpolation method (see Matlab documentation on `interp1` for further information)
- `extrapValue` - value with which the input is padded for extrapolation (if interpolation points are outside of the range $[1, n]$, n being the length of the input)

Components

- `output` - the result of the interpolation

Inputs The `Interpolation1D` element must have exactly one one-dimensional input.

5.5 Output Functions

5.5.1 Sigmoid

Description Computes the sigmoid (logistic function) of an input of arbitrary size and dimensionality. The output O is computed from the input I at every position as

$$O(x) = \frac{1}{1 + \exp(\beta(I(x) - \theta))} \tag{8}$$

with threshold θ and steepness parameter β. (Note: A logistic output function with threshold 0 is also implemented in the `NeuralField` class, so no separate element is required to compute the field output.)

Parameters

- `size` - size of the input and output of the element
- `beta` - steepness parameter of the logistic function
- `theta` - threshold for the logistic function

Components

- `output` - the sigmoid of the input

Inputs The `Sigmoid` element must have exactly one input whose size matches the element's `size` parameter.

5.5.2 HalfWaveRectification

Description Element that applies a positive half-wave rectification to the input x:

$$O(x) = \begin{cases} x & \text{if } x > 0 \\ 0 & \text{else} \end{cases} \tag{9}$$

This can be used as an alternative, non-saturating output function for neural fields or nodes.

Parameters

- `size` - size of the input and output of the element

Components

- `output` - the half-wave rectified input

Inputs The `HalfWaveRectification` element takes exactly one input whose size matches the element's `size` parameter.

5.6 Static Stimuli

5.6.1 BoostStimulus

Description A scalar stimulus that can be used as a homogenous boost of a neural field.

Parameters

- `amplitude` - value of the scalar stimulus

Components

- `output` - that same scalar value

5.6.2 GaussStimulus1D

Description A one-dimensional Gaussian.

Parameters

- `size` - size of the output
- `sigma` - width parameter of the Gaussian function
- `amplitude` - strength of the Gaussian stimulus
- `position` - position of the stimulus center
- `circular` - flag indicating whether the stimulus is defined in a circular fashion (i.e., if the stimulus is centered near the left end of the output, it can flow over into the right end and vice versa); default value is true
- `normalized` - flag indicating whether the Gaussian stimulus is normalized before scaling it with the amplitude (for a normalized stimulus, the integral is one if the amplitude is one; without normalization, the value at the center is one if the amplitude is one); default value is false

Components

- `output` - the Gaussian stimulus

5.6.3 GaussStimulus2D

Description A two-dimensional Gaussian.

Parameters

- `size` - size of the output
- `sigmaY` - width parameter of the Gaussian function along the vertical axis
- `sigmaX` - width parameter of the Gaussian function along the horizontal axis
- `amplitude` - strength of the Gaussian stimulus
- `positionY` - vertical position of the stimulus center
- `positionX` - horizontal position of the stimulus center
- `circularY` - flag indicating whether the stimulus is defined in a circular fashion in the vertical dimension (i.e., if the stimulus is center near the top end of the output, it can flow over into the bottom end and vice versa); default value is true

- `circularX` - flag indicating whether the stimulus is defined in a circular fashion in the horizontal dimension; default value is true
- `normalized` - flag indicating whether the Gaussian stimulus is normalized before scaling it with the amplitude (for a normalized stimulus, the integral is one if the amplitude is one; without normalization, the value at the center is one if the amplitude is one); default value is false

Components

- `output` - the Gaussian stimulus

5.6.4 GaussStimulus3D

Description A three-dimensional Gaussian.

Parameters

- `size` - size of the output
- `sigmaY`, `sigmaX`, `sigmaZ` - width parameters of the Gaussian function along each dimension
- `amplitude` - strength of the Gaussian stimulus
- `positionY`, `positionX`, `positionZ` - position of the stimulus center along each dimension
- `circularY`, `circularX`, `circularZ` - binary flags indicating whether the stimulus is defined in a circular fashion along each dimension (e.g., if the stimulus is centered near the top end of the output, it can flow over into the bottom end and vice versa); default value is true
- `normalized` - flag indicating whether the Gaussian stimulus is normalized before scaling it with the amplitude (for a normalized stimulus, the integral is one if the amplitude is one; without normalization, the value at the center is one if the amplitude is one); default value is false

Components

- `output` - the Gaussian stimulus

5.6.5 CustomStimulus

Description A freely defined stimulus pattern of arbitrary dimensionality. The full stimulus pattern is specified directly as a parameter. (Note that this can lead to large parameter files as the whole pattern needs to be stored.)

Parameters

- `size` - size of the output
- `pattern` - the full stimulus pattern (matching the `size` parameter)

Components

- `output` - the custom stimulus pattern

5.6.6 NormalNoise

Description Random noise drawn from a normal distribution in every time step.

Parameters

- `size` - size of the generated output
- `amplitude` - scalar value with which the noise is scaled

Components

- `output` - the generated matrix of scaled random values

5.7 Time-Dependent Stimuli and Switches

5.7.1 TimedSwitch

Description A connective element whose output is either zero or mirrors the input, with switches between these two states occuring at specified simulation times.

Parameters

- `size` - size of input and output
- `onTimes` - Nx2 matrix of the form `[tStart1, tEnd1; ...; tStartN, tEndN]`; the stimulus is active at simulation time t if $tStart_k <= t <= tEnd_k$ for any k

Components

- `output` - either the same as the input, or a matrix of zeros

Inputs The `TimedSwitch` element must have exactly one input whose size matches the element's `size` parameter.

5.7.2 TimedGaussStimulus1D

Description A one-dimensional Gaussian stimulus that is active at specified periods in simulation time.

Parameters

- `size` - size of the output
- `sigma` - width parameter of the Gaussian function
- `amplitude` - strength of the Gaussian stimulus
- `position` - position of the stimulus center

- `onTimes` - $N \times 2$ matrix of the form `[tStart1, tEnd1; ...; tStartN, tEndN]`; the stimulus is active at simulation time t if $tStart_k <= t <= tEnd_k$ for any k
- `circular` - flag indicating whether the stimulus is defined in a circular fashion (i.e., if the stimulus is centered near the left end of the output, it can flow over into the right end and vice versa); default value is true
- `normalized` - flag indicating whether the Gaussian stimulus is normalized before scaling it with the amplitude (for a normalized stimulus, the integral is one if the amplitude is one; without normalization, the value at the center is one if the amplitude is one); default value is false

Components

- `output` - the Gaussian stimulus

5.7.3 TimedGaussStimulus2D

Description A two-dimensional Gaussian stimulus that is active at specified periods in simulation time.

Parameters

- `size` - size of the output
- `sigmaY` - width parameter of the Gaussian function along the vertical axis
- `sigmaX` - width parameter of the Gaussian function along the horizontal axis
- `amplitude` - strength of the Gaussian stimulus
- `positionY` - vertical position of the stimulus center
- `positionX` - horizontal position of the stimulus center
- `onTimes` - Nx2 matrix of the form `[tStart1, tEnd1; ...; tStartN, tEndN]`; the stimulus is active at simulation time t if $tStart_k <= t <= tEnd_k$ for any k
- `circularY` - flag indicating whether the stimulus is defined in a circular fashion in the vertical dimension (i.e., if the stimulus is center near the top end of the output, it can flow over into the bottom end and vice versa); default value is true
- `circularX` - flag indicating whether the stimulus is defined in a circular fashion in the horizontal dimension; default value is true
- `normalized` - flag indicating whether the Gaussian stimulus is normalized before scaling it with the amplitude (for a normalized stimulus, the integral is one if the amplitude is one; without normalization, the value at the center is one if the amplitude is one); default value is false

Components

- `output` - the Gaussian stimulus

5.7.4 TimedCustomStimulus

Description A freely defined stimulus pattern of arbitrary dimensionality, which is active at specified periods in simulation time (and zero otherwise). The full stimulus pattern is specified directly as a parameter. (Note that this can lead to large parameter files as the whole pattern needs to be stored.)

Parameters

- `size` - size of the output
- `pattern` - the full stimulus pattern (matching the `size` parameter)
- `onTimes` - Nx2 matrix of the form `[tStart1, tEnd1; ...; tStartN, tEndN]`; the stimulus is active at simulation time t if $tStart_k <= t <= tEnd_k$ for any k

Components

- `output` - the custom stimulus pattern

5.7.5 CustomStimulusSequnce

Description A sequence of freely defined one- or two-dimensional stimulus patterns. Each pattern becomes active at a specified start time and remains active until it is replaced by the next pattern. If you want the last pattern to be turned off at a specified time, add an additional zero pattern to the end of the sequence. The full stimulus patterns are specified directly as a parameter. (Note that this can lead to large parameter files as the whole pattern needs to be stored.)

Parameters

- `size` - size of each stimulus pattern
- `stimulusPatterns` - matrix of custom stimulus patterns, either a $[K \times N]$ matrix of K one-dimensional stimuli with size N, or a $[M \times N \times K]$ matrix of K two-dimensional stimuli with size $[M \times N]$
- `startTimes` - optional vector of start times for the specified stimuli; the index of the active stimulus at simulation time t is determined as $\max(i|t >= startTime_i)$; values in startTimes must be monotonically increasing; if not specified, startTimes will be set to $1 : K$

5.8 History

5.8.1 History

Description Element that stores its input at specified times. A vector of simulation times $[t_1, ..., t_K]$ must be specified. The input to the element at those times is then stored in a $K \times N$ matrix if the input is a vector of size N, or in a $N \times M \times K$ matrix if the input is a matrix of size $N \times M$. (Note: No input is stored if no Euler step occurs exactly at a specified time t_i, e.g. if $t_i = 31.5$ and the step size of the simulation is 1, with steps occurring at $t = 31$ and $t = 32$.)

Parameters

- `size` - size of input (at one time step)
- `storingTimes` - vector of simulation times at which the input is stored

Components

- `output` - matrix of stored inputs (filled with NaN before inputs are stored)

Inputs The `History` element must have exactly one input whose size matches the element's `size` parameter.

5.8.2 RunningHistory

Description A continuously updated history of the input to this element over the recent time steps. At specified intervals, the input to this element is stored into a matrix (same format as in `History`) as first row/layer, while the content of all other rows/layers get pushed back. The oldest stored inputs are lost once the history matrix is full.

Parameters

- `size` - size of input (at one time step)
- `timeSlots` - number of time steps that are stored
- `interval` - interval between storing times (in simulation time; a new input is stored if simulation time modulo interval is zero)

Components

- `output` - matrix of stored inputs (filled with NaN before inputs are stored)

Inputs The `RunningHistory` element must have exactly one input whose size matches the element's `size` parameter.

5.9 Image Acquisition and Processing

5.9.1 CameraGrabber

Description Retrieves a new image from a connected camera in every step. Note: This class is specific for the cameras used at the INI and requires additional mex files to provide the actual link to the hardware.

Parameters

- `device` - device number for the connected camera (typically 0 or 1)
- `size` - size of the produced image; the image taken from the camera is resized if the sizes do not match

Components

- `image` - RGB image of size `[size(1), size(2), 3]`

5.9.2 ImageLoader

Description Loads images from file, allows switching between different images.

Parameters

- `fileNames` - cell array of file names; in the constructor a separate string `filePath` can be provided as common path to the image files
- `size` - size of output image; loaded images are resized if their size does not match this value
- `currentSelection` - index of the currently selected image file

Components

- `image` - RGB image of size `[size(1), size(2), 3]`

5.9.3 ColorExtraction

Description Extracts regions of salient color information from an RGB image. The steps of the color extraction are as follows:

- the region of interest is horizontally resized to match the specified horizontal output size (the vertical size of the region of interest remains unchanged), and transformed into HSV color space
- pixels are determined that exceed a specified saturation and value threshold, and classified according to their hue value into a number discrete colors
- the pixels are summed up vertically to determine for each horizontal position the number of salient pixels of each color

The result is a matrix of size `[nColors, NX]`, where each row yields the distribution of salient color pixels over the horizontal axis of the region of interest.

Parameters

- `roi` - region of interest in the image, within which regions of salient color are extracted; in the constructor, this region is defined by two arguments `imageRangeX` and `imageRangeY`, giving the horizontal and vertical extent of the roi as a two-element vector in pixels of the input image
- `size` - size of the produced matrix, with `size = [nColors, NX]`
- `hueToIndexMap` - matrix specifying which ranges of hue values are mapped onto which discrete colors; the matrix has the form

  ```
  [hueMin_1, hueMax_1, colorIndex_1; ...
   hueMin_2, hueMax_2, colorIndex_2; ...]
  ```

  where each `[hueMin, hueMax]` pair defines a range in hue values, and `colorIndex` is the row in the output matrix to which this color range is counted; multiple ranges of hue values may be mapped to a single `colorIndex`; the total range of hue values is [0, 1]

- `saturationThreshold` - saturation threshold in HSV color space for a pixel to be counted toward any of the colors; the total range of saturation values is [0, 1]
- `valueThreshold` - value threshold in HSV color space a for pixel to be counted toward any of the colors; the total range of value values is [0, 1]

Components

- `output` - matrix of salient color distributions

Inputs The `ColorExtraction` element takes as input one RGB image (matrix of size [M, N, 3]). The specified `roi` has to be within the bounds of this image.

5.10 Motor control

5.10.1 AttractorDynamics

Description Forms a one-dimensional dynamical system from a space-coded input and determines the rate of change for a given state of the system. The dynamical system is defined by scaling shifted sigmoid functions with the space-coded input, producing attractors in regions of high input.

Parameters

- `size` - size of the one-dimensional space-coded input
- `amplitude` - amplitude or scaling factor for the output

Components

- `phiDot` - rate of change for the given state of the dynamical system
- `phiDotAll` - vector of the same size of the input, giving the rate of change for all possible states of the dynamical system

Inputs The `AttractorDynamics` element takes exactly two inputs: The first is a one-dimensional space code whose size matches the element's `size` parameter, the second is a scalar value that specifies the current state of the dynamical system.

5.10.2 DynamicRobotController

Description Connects the DNF architecture to an E-Puck robot (requires additional files for the interface) and allows dynamic control of the robot orientation by specifying its rate of change.

Parameters

- `minWheelVelocity` - minimal velocity for both wheels (velocity is set to zero if absolute value of derived from the orientation dynamics is lower than this value); avoids poor odometry due to erratic robot movements
- `maxWheelVelocity` - maximum velocity for both wheels

Components

- `position` - robot position relative to its starting position (determined from odometry)
- `orientation` - robot orientation, given in the range $[-\pi, \pi)$

Inputs The `DynamicRobotController` takes exactly one input that defines the rate of change for the robot orientation (in rad per second).

5.10.3 MobileRobotArena

Description Provides a simulation of a mobile robot moving in an arena. Inputs to the element control the movement of the robot. The robot can detect targets in the arena with an array of directed sensors. The element provides as components the robot's position (a two-element vector), its orientation, the raw values of the sensor array, and these values projected onto the orientation space (with a Gaussian profile of fixed width scaled with the sensor output for each sensor). Number and direction of sensors can be adjusted by manually changing the parameter `sensorAngles` (requires initalization).

Parameters

- `arenaSize` - size of simulated rectangular arena (a two-element vector)
- `robotSize` - diameter of the simulated cylindrical robot
- `targetSize` - diameter of the simulated sensor targets
- `robotPoseInit` - initial pose of the robot (three-element vector specifying position and orientation)
- `sensorAngles` - angular positions of the sensors on the cylindrical robot; this parameter also determines the number of simulated sensors
- `sigmaSensor` - measure for the sensors' opening angle
- `sensorOutputSize` - size of the component `sensorOutput` (see below)
- `sigmaOutput` - width parameter of the Gaussian that each sensor contributes to the component `sensorOutput`
- `forwardSpeed` - forward speed of the robot, or scaling factor of the forward speed if the element receives two inputs
- `sensorNoiseLevel` - level of random noise added to the sensor responses
- `sensorOutputZeroPosition` - position of the robot's forward direction when angular space is mapped to vector `sensorOutput`

Components

- `robotPosition` - the current position of the robot (as two-element vector)
- `robotPhi` - the current orientation of the robot
- `rawSensorValues` - vector of sensor responses, with one entry for each sensor
- `sensorOutput` - vector that can be used to feed the sensor responses as input into a field; the vector samples angular space; for each sensor, a Gaussian profile of fixed width `sigmaOutput` is created centered on the angular position of the sensor; this Gaussian profile is scaled with response of the sensor

Inputs The element can receive either one or two inputs: The first one is the rate of change of the robot's heading direction; the optional second one is a forward speed. If the second input is provided, it is scaled with the parameter `forwardSpeed`, otherwise `forwardSpeed` is taken directly as the robot's speed.

6 Controls

6.1 ParameterSlider

The `ParameterSlider` control creates a slider with an accompanying text field in the GUI. The slider is connected to one or more parameters (belonging to a single element or different elements). The parameter value is changed whenever the slider is moved. The range of parameter values covered by the slider as well as a scaling factor for the conversion from slider position to parameter value can be specified.

The control handle is obtained via the constructor call

```
ParameterSlider(controlLabel, elementLabels, parameterNames, ...
  sliderRange, valueFormat, scalingFactor, toolTip, position)
```

with the following arguments:

- `controlLabel` - label for the control displayed in the text field next to the slider
- `elementLabels` - string or cell array of strings specifying the labels of elements controlled by this slider
- `parameterNames` - string or cell array of strings specifying the names of the element parameters controlled by this slider; arguments `elementLabels` and `parameterNames` must have the same size, with each pair of entries fully specifying one controlled parameter
- `sliderRange` - two-element vector giving the range of the slider
- `valueFormat` - string specifying the format of the parameter value displayed next to the slider (optional, see the Matlab documentation of the `fprintf` function on construction of that string)
- `scalingFactor` - scalar value specifying a conversion factor from the element's parameter value to the slider position (optional)
- `tooltip` - tooltip displayed when hovering over the control with the mouse (optional)

- `position` - position of the control in the GUI figure window in relative coordinates (optional, is overwritten when specifying a grid position in the GUI's `addControl` function)

The slider position is initialized to reflect the value of the first connected parameter and is adjusted when this parameter value is changed via any other control (within the range of the slider).

Example

```
h = ParameterSlider('h_u', 'field u', 'h', [-10, 0], '%0.1f', 1,
  'resting level of field u');
```

6.2 ParameterSwitchButton

The `ParameterSwitchButton` control creates a labeled toggle button (i.e. the button toggles between pressed and not pressed when clicked). This control can switch the values of one or more parameter between two predefined values. Note: The state of the button is not adjusted when the controlled parameters are changed via another control or the parameter panel. The button's state does therefore not necessarily reflect the current parameter values.

The control handle is obtained via the constructor call

```
ParameterSwitchButton(controlLabel, elementLabels, parameterNames,
  offValues, onValues, toolTip, pressedOnInit, position)
```

with the following unique arguments (see above for a description of the other arguments):

- `offValues` - scalar or vector specifying for every connected element parameter the value it should take while the button is not pressed
- `onValues` - scalar or vector specifying for every connected element parameter the value it should take while the button is pressed
- `pressedOnInit` - specifies whether the button should be in the pressed or not pressed state on initialization of the GUI (default is false)

Example

```
h = ParameterSlider('h_u', 'field u', 'h', [-10, 0], '%0.1f', 1,
  'resting level of field u');
```

6.3 ParameterDropdownSelector

The `ParameterDropdownSelector` control creates a dropdown menu with an accompanying text field in the GUI. The menu allows to change the values of one or more parameters between a number of presets.

The control handle is obtained via the constructor call

```
ParameterDropdownSelector(controlLabel, elementLabels, ...
  parameterNames, dropdownValues, dropdownLabels, ...
  initialSelection, toolTip, position)
```

with the following unique arguments (see above for a description of the other arguments):

- dropdownValues - a numerical array or a cell array of numerical arrays specifying the parameter values associated with each menu entry; if the control is connected to a single element parameter, this argument should be an array with one valid parameter value for each item in the dropdown menu; if multiple parameters are connected, it should be a cell array of such arrays
- dropdownLabels - cell array of strings specifying the menu items in the dropdown menu (optional, if not specified the dropdownValues for the first connected parameter are used as labels)
- initialSelection - integer specifying the initial selection in the dropdown menu (optional, default is 1)

Examples

```
h = ParameterDropdownSelector('p_sA', 'stimulus A', 'position', ...
  [25, 50, 75], {'left', 'center', 'right'}, 2, ...
  'position of stimulus A');
h = ParameterDropdownSelector('d_s', {'stimulus A', 'stimulus B'},...
  {'position', 'position'}, {[40, 45, 48], [60, 55, 52]}, ...
  {'far', 'close', 'very close'}, 1, 'distance between stimuli A and B');
```

6.4 GlobalControlButton

The GlobalControlButton control creates a button in the GUI that connects to a property of any specified object, typically the GUI itself. For the StandardGUI, it can connect to one of the following boolean properties: pauseSimulation, quitSimulation, resetSimulation, saveParameters, loadParameters, and paramPanelRequest. The values of these flags are checked in every cycle of the GUI and appropriate operations will be performed.

The control handle is obtained via the constructor call

```
GlobalControlButton(controlLabel, controlledObject, ...
  propertyName, onValue, offValue, resetAfterPress, ...
  toolTip, position)
```

with the following unique arguments (see above for a description of the other arguments):

- controlledObject - handle to the controlled object
- propertyName - property name that is controlled by the button
- onValue - value that the controlled property should have while the button is pressed
- offValue - value that the controlled property should have while the button is not pressed
- resetAfterPress - determines the behavior of the button: for false it acts as a toggle button, for true as a push button; in the latter case, the controlled property will always be set to the onValue when the button is clicked

Examples

```
h = addControl(GlobalControlButton('Pause', gui, 'pauseSimulation', ...
  true, false, false, 'pause simulation'));
```

6.5 PresetSelector

The `PresetSelector` control loads full parameter settings for the model from one of a set of predefined parameter files. The control consists of a dropdown menu to select a parameter file (which may be listed with a descriptive label) and a confirmation button. When the button is pressed, the parameter file connected to the currently selected entry in the dropdown menu is loaded.

Note: Loading from a parameter file will re-initialize the simulation. The control handle is obtained via the constructor call

```
PresetSelector(controlLabel, controlledObject, filePath, presetFiles,
  presetLabels, toolTip, position)
```

with the following unique arguments (see above for a description of the other arguments):

- `controlledObject` - the object that performs the loading operation, which is always the GUI that the control is part of
- `filePath` - string specifying a common relative or absolute path for all parameter files (may be empty if files are located in different folders)
- `presetFiles` - cell array of strings containing the file names (or complete paths) for the parameter files
- `presetLabels` - cell array of strings containing a label for each parameter file to be shown in the dropdown menu (optional, by default the filenames are used as labels)

Example

```
h = PresetSelector('Select', gui, 'presetsOneLayerField/', ...
  {'stabilized.json', 'selection.json', 'memory.json'}, ...
  {'stabilized', 'selection', 'memory'}, ...
  'Load pre-defined parameter settings');
```

6.6 RobotArenaControlPanel

Control element specifically designed to control and visualize a `MobileRobotArena` element. The panel opens in separate window, showing a top-down view of a robot arena with a mobile robot and sensor targets. Targets can be added or removed using the provided buttons.

The control handle is obtained via the constructor call

```
RobotArenaControlPanel(elementLabel, figurePosition, figureTitle)
```

with the following arguments:

- `elementLabel` - label of a `MobileRobotArena` element
- `figurePosition` - position of the figure window on the screen
- `figureTitle` (optional) - title displayed on the figure window

Example

```
h = RobotArenaControlPanel('arena', [500, 50, 600, 720]);
```

7 Visualizations

7.1 MultiPlot

The `MultiPlot` visualization creates a set of axes with one or more plots in it, oriented either vertically or horizontally.

The visualization handle is obtained via the constructor call

```
MultiPlot(plotElements, plotComponents, scales, orientation, ...
  axesProperties, plotProperties, title, xlabel, ylabel, position)
```

with the following arguments:

- `plotElements` - string or cell array of strings (with one entry for each plot) listing the labels of the elements whose components should be plotted
- `plotComponents` - string or cell array of strings (with one entry for each plot) listing the component names that should be plotted for the specified elements; one pair of entries from `plotElements` and `plotComponents` fully specifies the source data for one plot
- `scales` - scalar or numeric vector specifying a scaling factor for each plot (optional, by default all scaling factors are 1)
- `orientation` - string specifying the orientation of the plot, should be either `horizontal` (default) or `vertical`; for horizontal plots, the component is used as `YData` of the plot, for vertical plots it is used as `XData`; the data for the respective other axis is fixed and can be set in the `plotProperties` argument
- `axesProperties` - cell array containing a list of valid axes settings (as property/value pairs) that can be applied to the axes handle via the `set` function (optional, see Matlab documentation on `axes` for further information)
- `plotProperties` - cell array of cell arrays containing lists of valid lineseries settings (as property/value pairs or as a single string specifying the line style) that can be applied to the plot handles via the `set` function (see Matlab documentation on the `plot` function for further information); the outer cell array must contain one inner cell array for every plot (optional)
- `title` - string specifying an axes title (optional)
- `xlabel` - string specifying an x-axis label (optional)
- `ylabel` - string specifying a y-axis label (optional)
- `position` - position of the control in the GUI figure window in relative coordinates (optional, is overwritten when specifying a grid position in the GUI's `addVisualization` function)

It is also possible to create a `MultiPlot` object without specifying what is to be plotted (giving empty matricies for arguments in the constructor call that refer to individual plots), and then add plots individually using the `addPlot` function. However, this can only be done *before* the visualization is added to the GUI. The sequence of function calls then has the following form:

```
hMP = MultiPlot([], [], [], 'horizontal', {...});
hMP.addPlot(...);
hMP.addPlot(...);
gui.addVisualization(hMP, ...);
```

The method call for adding plots has the form

```
hMP.addPlot(plotElement, plotComponent, plotProperties, scale)
```

with arguments

- `plotElement` - label of the element whose component should be plotted
- `plotComponent` - name of the element component that should be plotted
- `plotProperties` - cell array containing a list of valid lineseries settings (as property/value pairs or as a single string specifying the line style) that can be applied to the plot handle via the `set` function (optional, see Matlab documentation on the `plot` function for further information)
- `scale` - scaling factor for the plot

Example

```
h = MultiPlot({'field u', 'field u', 'stimulus A'}, ...
  {'activation', 'output', 'output'}, [1, 10, 1], 'horizontal', ...
  {'YLim', [-10, 10]}, { {'b-', 'LineWidth', 2}, {'r-'}, {'g--'} }, ...
  'perceptual field', 'feature value', 'activation');
```

7.2 XYPlot

The `XYPlot` visualization creates a set of axes with one or more plots in it. The difference to `MultiPlot` is that here, both the `XData` and `YData` of each individual plot can be specified as either the component of some element, or as a fixed vector. This makes it possible to plot two components against each other.

The visualization handle is obtained via the constructor call

```
XYPlot(plotElementsX, plotComponentsOrDataX, ...
  plotElementsY, plotComponentsOrDataY, ...
  axesProperties, plotProperties, title, xlabel, ylabel, position)
```

with the following arguments:

- `plotElementsX` - cell array with one entry for each plot: either the label of the element (as string) whose component should be used as `XData` for the plot, or an empty array `[]` if the `XData` is to be a fixed vector (if only a single plot is created, the cell array may be omitted in this and the following arguments)
- `plotComponentsOrDataX` - cell array with one entry for each plot: either the component name (as string) that should be plotted for the specified element, or the fixed vector that should be used as `XData` for that plot
- `plotElementsY` - cell array specifying the source elements for the plots' `YData`, analogous to the parameter `plotElementsX`

- `plotComponentsOrDataY` - cell array specifying the element components or fixed vectors to be used as `YData`, analogous to the parameter `plotComponentsOrDataY`
- `axesProperties` - cell array containing a list of valid axes settings (as property/value pairs) that can be applied to the axes handle via the `set` function (optional, see Matlab documentation on `axes` for further information)
- `plotProperties` - cell array of cell arrays containing lists of valid lineseries settings (as property/value pairs or as a single string specifying the line style) that can be applied to the plot handles via the `set` function (see Matlab documentation on the `plot` function for further information); the outer cell array must contain one inner cell array for every plot (optional)
- `title` - string specifying an axes title (optional)
- `xlabel` - string specifying an x-axis label (optional)
- `ylabel` - string specifying a y-axis label (optional)
- `position` - position of the control in the GUI figure window in relative coordinates (optional, is overwritten when specifying a grid position in the GUI's `addVisualization` function)

As in `MultiPlot`, it is also possible to first create the visualization without specifying the sources and properties of the individual plots (placing empty matrices for the arguments in the constructor), and then to add plots individually through the function `addPlot`:

```
hXYP = XYPlot({}, {}, {}, {}, axesProperties, {});
hXYP.addPlot(plotElementX, plotComponentOrDataX, ...
  plotElementY, plotComponentOrDataY, plotProperties)
```

Again, plots can only added *before* the visualization object itself is added to the GUI.

Example

```
h = XYPlot('node u', 'activation', 'node v', 'activation', ...
  {'XLim', [-10, 10], 'YLim', [-10, 10], 'Box', 'on'}, ...
  { {'bo', 'MarkerSize', 5} }, ...
  'phase plot', 'activation u', 'activation v');
```

7.3 SlicePlot

The `SlicePlot` visualization plots one-dimensional slices (rows or columns) taken at specified positions from one or several two-dimensional input matries.

The visualization handle is obtained via the constructor call

```
SlicePlot(plotElements, plotComponents, plotSlices, ...
  sliceOrientations, scales, plotOrientation, ...
  axesProperties, plotProperties, ...
  title, xlabel, ylabel, position)
```

with the following unique arguments (see above for a description of the other arguments):

- `plotElements` - string or cell array of strings listing the labels of the elements from which slices should be plotted

- `plotComponents` - string or cell array of strings listing the component names from which slices should be plotted for the specified elements; one pair of entries from `plotElements` and `plotComponents` fully specifies the source data for one set of slice plots

- `plotSlices` - integer vector or cell of integer vector, specifying for each entry in `plotElements` a set of indices of the rows or columns that should be plotted

- `sliceOrientations` - string or cell array of strings with each entry either 'horizontal' or 'vertical', specifying the slice orientation (rows or columns) for each entry in `plotElements`

- `scales` - scalar or numeric vector specifying a scaling factor for each set of slices (optional, by default all scaling factors are 1)

- `plotOrientation` - string specifying the orientation of all plots, should be either `horizontal` (default) or `vertical`; for horizontal plots, the slices are used as `YData` of the plot, for vertical plots they are used as `XData`; the data for the respective other axis is fixed and can be set in the `plotProperties` argument

Example

```
h = SlicePlot('field u', 'activation', [25, 50, 75], 'horizontal', ...
   1, 'horizontal', {'YLim', [-10, 10]}, {{'r-'}, {'g-'}, {'b-'}}, ...
  'three slices through field u', 'field position', 'activation');
```

7.4 ScaledImage

The `ScaledImage` visualization plots two-dimensional data using the Matlab `imagesc` function. The visualization handle is obtained via the constructor call

```
ScaledImage(imageElement, imageComponent, imageRange, axesProperties, ...
  imageProperties, title, xlabel, ylabel, position)
```

with the following unique arguments (see above for a description of the other arguments):

- `imageElement` - label of the element whose component should be visualized

- `imageComponent` - name of the element component that should be plotted

- `imageRange` - two-element vector specifying the range of the image's color code

- `imageProperties` - cell array containing a list of valid image object settings (as property/value pairs) that can be applied to the image handle via the `set` function (optional, see Matlab documentation on the `image` function for further information)

Example

```
h = ScaledImage('field u', 'activation', [-10, 10], ...
  {'YDir', 'normal'}, {}, 'perceptual field', 'position', 'color');
```

7.5 RGBImage

The RGBImage visualization interprets a 3xMxN matrix as an RGB image and displays it via the Matlab image function. The visualization handle is obtained via the constructor call

```
RGBImage(RGBImage(imageElement, imageComponent, axesProperties, ...
  imageProperties, title, xlabel, ylabel, position))
```

(see above for a description of the arguments).

Example

```
h = RGBImage('camera grabber', 'output', {'YDir', 'normal'}, {}, ...
  'camera image');
```

7.6 SurfacePlot

The SurfacePlot visualization diplays two-dimensional data either as a mesh or a surface plot. The visualization handle is obtained via the constructor call

```
SurfacePlot(plotElement, plotComponent, zLim, plotType, ...
  axesProperties, plotProperties, ...
  title, xlabel, ylabel, zlabel, position)
```

with the following unique arguments (see above for a description of the other arguments):

- zLim - range of the plot's axes in the z-dimension
- plotType - either 'mesh' or 'surface' (default)
- plotProperties - cell array containing a list of valid surface object settings (as property/value pairs) that can be applied to the surface handle via the set function (optional, see Matlab documentation on the surface function for further information)
- zlabel - string specifying a z-axis label (optional)

Example

```
h = SurfacePlot('field u', 'activation', [-10, 10], {}, {}, ...
  'perceptual field', 'color', 'position', 'activation');
```

7.7 KernelPlot

The KernelPlot visualization is used to plot an interaction kernel. It can combine different kernels and global interaction strengths into a single correctly aligned plot: Local interaction kernels are all centered at zero along the x-axis, capped or padded with zeros as necessary on both sides to match the specified kernelRange for the plot, and added up. Global components of interaction kernels are added globally to the plot. The visualization handle is obtained via the constructor call

```
KernelPlot(plotElements, plotComponents, kernelTypes, plotRange, ...
  axesProperties, plotProperties, title, xlabel, ylabel, position)
```

with the following unique arguments (see above for a description of the other arguments):

- `plotElements` - string or cell array of strings specifying the elements that contribute to the plotted interaction kernel
- `plotComponents` - string or cell array of strings (with one entry for each plot) listing the component names for the specified elements; one pair of entries from `plotElements` and `plotComponents` fully specifies one contribution to the plotted interaction kernel
- `kernelTypes` - cell array of strings, with one entry of either `'local'` or `'global'` for each entry in `plotElements`
- `plotRange` - scalar value determining to which range (positively and negatively from zero) the kernel should be plotted
- `plotProperties` - cell array containing a list of valid lineseries settings (as property/value pairs or as a single string specifying the line style) that can be applied to the plot handle via the `set` function (optional, see Matlab documentation on the `plot` function for further information)

Example

```
h = KernelPlot({'u -> u', 'u -> u'}, {'kernel', 'amplitudeGlobal'}, ...
  {'local', 'global'}, 50, {'YLim', [-1, 1]}, {'r-', 'LineWidth', 2}, ...
  'kernel plot', 'distance in feature space', 'interaction weight');
```

7.8 StaticText

The `StaticText` visualization displays a text in the figure window. The constructor call is

```
StaticText(text, position)
```

with the following arguments:

- `text` - text to be displayed (as string)
- `position` - position of the control in the GUI figure window in relative coordinates (optional, is overwritten when specifying a grid position in the GUI's `addVisualization` function)

Example

```
h = StaticText('This field receives no external input.');
```

To place this visualization in the controls grid of a GUI:

```
gui.addVisualization(StaticText('Stimulus controls'), ...
  [1, 1], [1, 1], 'control');
```

7.9 TimeDisplay

The `TimeDisplay` visualization prints the current simulation time. The constructor call is

```
TimeDisplay(caption, units, valueFormat, position)
```

with the following arguments:

- `caption` - string printed before the simulation time (optional, default is 'Simulation time: ')
- `units` - string printed after the simulation time (optional, empty by default)
- `valueFormat` - string specifying the number format in which the simulation time is displayed (optional, see the Matlab documentation of the fprintf function for help on constructing that string)
- `position` - position of the control in the GUI figure window in relative coordinates (optional, is overwritten when specifying a grid position in the GUI's `addVisualization` function)

Example

```
h = TimeDisplay('Time: ', ' s', '%0.1f');
```

To place this visualization in the controls grid of a GUI:

```
gui.addVisualization(TimeDisplay(), [1, 1], [1, 1], 'control');
```

Part III
Expanding the Framework

8 Overview

The COSIVINA framework is intended to support easy expansion by its users to meet their specific needs. This expansion is achieved by adding new classes for elements, controls, or visualizations. If these classes match the formats and specifications used in the framework, they can easily be integrated into existing architectures and GUIs. The following sections describe how new classes should be designed and organized to be used with COSIVINA.

9 Creating New Element Classes

9.1 General Structure

Each element class in the COSIVINA architecture is derived from the abstract base class `Element` and has the following general form:

```
% ExampleElement (COSIVINA toolbox)
%   Please provide a description of the element.

classdef ExampleElement < Element
  properties (Constant)
    parameters = struct('size', ParameterStatus.Fixed, ...);
    components = {'output', ...};
    defaultOutputComponent = 'output';
  end

  properties
    % parameters
    size
    ...

    % accessible structures
    output
    ...

    % internal variables
    ...
  end

  methods
    function obj = ExampleElement(label, size, ...)
      % constructor
    end

    function obj = init(obj)
      % perform intialization
```

```
    end

    function obj = step(obj, time, deltaT)
      % perform step
    end

    function obj = close(obj)
      % close any external connections (optional)
    end
  end
end
```

Each element class must have constant properties `parameters`, `componenent`, and `defaultOutputComponent`, which provide meta information about this element for the `Simulator` and `StandardGUI` classes (e.g. for checking the consistency of connections between elements, saving/loading configuration files, or automatically creating entries in the parameter panel). Then it must define the listed parameters and components as actual properties, as well as any other internal variables needed in the class. Each element should provide a constructor, and must implement the functions `init` and `step`. A `close` function may be implemented if required.

All other functionality (like managing the connections between elements) is provided by the abstract superclass `Element`.

9.2 Parameters

The parameters are those properties of an element that are saved in configuration files and that can be changed by GUI controls. They should fully specify the behavior of an element (but not its current state) in a compact form. Each parameter must exist as a public property of the element (it must be public to allow setting the value when loading from a configuration file). Additionally, an entry with the parameter name must exist in the constant `parameters` struct. Here, a status must be specified for each parameter that describes whether the parameter may be changed and what actions are required for the parameter change to take effect. This is shown for a parameter `size` in the example above.

The class `ParameterStatus` defines four possible status values:

- `Fixed`: The parameter value is fixed and cannot easily be changed in a running simulation. This is the case e.g. for the `size` parameters of most elements, which must be kept in alignment with the sizes of other, connected elements. Fixed parameters may still be changed manually.

- `Changeable`: The parameter can be changed at any time and the change will take effect the next time the step function is called. This is the case e.g. for amplitude parameters that are multiplied with the input in any step, see e.g. the element `ScaleInput`.

- `InitRequired`: The element must be re-initialized for the parameter change to take effect (and the element will be in the appropriate state for the running simulation after the initilization). This is the case if a parameter is not used directly in the step function, but is used e.g. for computing the value of an internal variable during initialization. It is also appropriate in constant stimulus elements, where the output is created only during intialization (and the step function is empty), see e.g. `GaussStimulus1D`.

- `InitStepRequired`: The element must be re-initialized for the parameter change to take effect, but the elements components may not be in the appropriate state for the running simulation after this initialization. The step function must be called again to update the components. This situation arises e.g. in the `GaussKernel1D` element: If an interaction parameter is changed, the element must be re-initialized to update the interaction kernel (stored internally as a vector). This also resets the elements output to zero. This incorrect output might be used in the next simulation step before the element's step function is called. To avoid this, the element's step function is called manually after a parameter change.

There is an additional set of status values that specify the size constraints for non-scalar parameters:

- `FixedSizeMatrix`: The parameter is a matrix of fixed size.
- `VariableRowsMatrix`: The parameter is a matrix whose number of rows may be changed during simulation. This is used e.g. in the class `TimedGaussStimulus1D`, where an $n \times 2$ matrix specifies the times at which a stimulus is active.
- `VariableColumnsMatrix`: The parameter is a matrix whose number of columns may be changed during simulation.
- `VariableSizeMatrix`: The number of both rows and columns may be changed during simulations.

The parameter status is implemented as an integer value interpreted as a string of bits, each bit yielding one piece of information. To specify that a parameter is matrix of any type, combine one entry from the first set with an entry from the second set using the function `bitor`, e.g.:

```
bitor(ParameterStatus.Changeable, ParameterStatus.VariableRowsMatrix}
```

The parameter status values are used to take the appropriate action in the method `setElementParameters` of the `Simulator` class. This method is also used by the GUI controls to effect a change in a parameter value.

9.3 Components

The components are those properties of an element that can serve as input to another element or be visualized in the GUI. Similar to the parameters, each component must exist as a property and its name must exist as an entry in the constant `components` property (a cell array of strings). Additionally, one component name must be specified as `defaultOutputComponent`, which is used when no component is specified for a connection between two elements. This is shown for a component `output` in the example above. Note that a property may be a parameter and a component at the same time (e.g. the resting level `h` in the `NeuralField` class).

9.4 Constructor

Each element should provide a constructor for easy creation of instances of that class. For loading elements from configuration files, it is necessary that the constructor can be called without any arguments (e.g. by checking for `nargin > 0` and setting default values if no arguments are provided). The first argument should be the element label (this property is

defined in the superclass `Element` and therefore exists in every derived class), the following arguments should specify the element parameters (though they do not need to match the actual parameters in name or format). After the constructor has been called, all element parameters should have valid values. The element components need not be initialized in the constructor.

9.5 Initialization

Each element class must have an `init` function with no arguments except for the class handle itself (called `obj` in the code above), and returning that class handle. This function is called at the beginning of each simulation, and may be called again after a parameter change (see above). Its purpose is set the element components to a state in which they may be accessed and used in the step functions of other elements. This means in particular that it must be ensured that all component variables are vectors/matrices of the correct size, so that other elements can perform computations on them (like adding components of different elements) without causing errors. The `init` function itself should not attempt to access the components of any other elements, since it cannot be guaranteed that those elements are already initialized.

The `init` function may also be used to perform preparatory computations that should not be repeated in every step (like computing the interaction kernel in the `GaussKernel1D` class). If this is done, set the status of the involved element parameters accordingly (see above). The function body may be empty if no initialization procedures are required for an element.

9.6 Simulation Step

Each element class must provide a `step` function with three arguments: the class handle, the current simulation time, and the amplitude of the time step. For many elements, the arguments `time` and `deltaT` are not needed and can be ignored. The step function is called once for every element in a simulator in every time step of the simulation. The order in which element step functions are called is determined by the order in which elements have been added to the simulator. The step function should update the element's components. Its function body may be empty if the components are static as long as parameters remain unchanged (see e.g. `GaussStimulus1D`).

The function may access the components of other elements that this element receives input from as `obj.inputElements{i}.(obj.inputComponents{i})`. Here, `inputElements` is a cell array of element handles and `inputComponents` is a cell array of strings, containing component names. Both are properties of the superclass `Element`, and are set appropriately if a new connection is defined. The step function may furthermore access the property `obj.nInputs` of the superclass to iterate over all inputs.

9.7 Close External Connections

If an element provides a connection to external programs, files, or hardware, it may be desirable to close these connections upon termination of a simulation. The `close` function of each element is called by the `close` function of the `Simulator` class. It does not have to be implemented if no closing or clean-up operations are required.

10 Creating New Control Classes

Controls are parts of a GUI that can be used to change element parameters or other simulation variables. A typical control class will create a graphical element such as a button or slider in the main figure window of a GUI, and affect the parameters of one or more elements in the connected simulator. However, none of these things are strictly required, and a control may behave differently (like creating a new figure window or affecting the GUI object itself). The descriptions here are therefore held rather general. You may want to look at existing control classes as guideline to create your own classes.

All controls are derived from the abstract base class `Control` and have the following general form:

```
% ExampleControl (COSIVINA toolbox)
%   Please provide a description of the control.

classdef ExampleControl < Control
  properties
    % class properties
  end

  methods
    function obj = ExampleControl(..., position)
      % constructor
    end

    function obj = connect(obj, simulatorHandle)
      % connect to simulator object
    end

    function obj = init(obj, figureHandle)
      % initialize control object
    end

    function changed = check(obj)
      % check control object and update simulator object if required
    end

    function obj = update(obj)
      % update control object
    end
  end
end
```

Each control class must implement the `connect`, `init`, `check`, and `update` methods.

10.1 Constructor

Each control class should have a constructor to provide an easy way for adding that control to a GUI. The existing control classes all have an optional last argument `position` to set the property of the same name defined in the superclass `Control`. This argument allows the user to explicitly specify a relative position of the control in the figure window. Alternatively, the

property is set by the addControl method of the StandardGUI class to position the control in the controls grid. You may ignore that property if not applicable for the type of control you have in mind.

10.2 Connecting the Control to a Simulator

The function connect is used to establish a connection between a control and the simulator object, or the specific elements that are accessed by the control. This is done in a separate function such that an existing GUI may be used with a different simulator object than initially specified. This function should check that the elements and parameters that have been specified for the control do actually exist in the simulator, and a way to access them should be retained in the class's internal properties (e.g. as a handle to an element). If a control does not actually access the simulator object or an element in it, the body of this function may remain empty. You may also want to define a property that keeps track of whether the control is currently connected to a simulator or not.

10.3 Initialization

In the function init, the graphical elements for the control object should be created (typically in the GUI main figure window, a handle to which is provided as argument when this function is called by the GUI class). The control should be rendered ready to use after the init function has been called.

10.4 Check Control

The function check is called for each control in each GUI step. In this function, it should be determined whether the control has been used (e.g. button pressed / slider position altered). If this is the case, the appropriate change should be applied to the element parameters or other values that are connected to the control object. In most cases, this can be done most efficiently by calling the method setElementParameters of the Simulator object. The check function should return a boolean value that specifies whether the control has been used. This signals to the GUI that element parameters may have been altered, and that it may be necessary to update the other controls (and possibly the parameter panel) to reflect these changes.

10.5 Update Control

The function update is used to update the state of a graphical control element when a change of the controlled parameter value may have occurred due to the use of another control. For instance, the position of a parameter slider is updated to reflect the current parameter value after that has changed due to an action in another control or in the parameter panel. If your control should not reflect any changes in the controlled parameters, the body of this function may remain empty.

11 Creating New Visualization Classes

Visualizations are graphical elements that display aspects of the running simulation, typically element components, but do not actively affect the simulation in any way. Visualization classes are derived from the abstract superclass Visualization and have the following general form:

```
% ExamplePlot (COSIVINA toolbox)
%   Please provide a description of the visualization.

classdef ExamplePlot < Visualization
  properties
    % visualization properties
  end

  methods
    function obj = ExamplePlot(..., position)
      % constructor
    end

    function obj = connect(obj, simulatorHandle)
      % connect to simulator object
    end

    function obj = init(obj, figureHandle)
      % initialization
    end

    function obj = update(obj)
      % update
    end
  end
end
```

Visualizations work largely analogous to control classes, but they do not provide a `check` function, and their `update` function is called in every GUI step.

11.1 Contructor

Each visualization class should have a constructor to provide an easy way for adding it to a GUI. The existing visualization classes all have an optional last argument `position` to set the property of the same name defined in the superclass `Visualization`. This argument allows the user to explicitly specify a relative position of the visualization in the figure window. Alternatively, the property is set by the `addVisualization` method of the `StandardGUI` class to position the visualization in the visualizations grid.

11.2 Connecting the Visualization to a Simulator

The function `connect` is used to establish a connection between a visualization and the simulator object, or the specific elements components that are displayed by the visualization. This is done in a separate function such that an existing GUI may be used with a different simulator object than initially specified. This function should check that the elements and components that have been specified for the visualization do actually exist in the simulator. It should then retain a way to access these components in the class's internal properties (e.g. as a handle to an element). You may also want to define a property that keeps track of whether the control is currently connected to a simulator or not.

11.3 Initialization

In the function `init`, the graphical elements for the visualization object should be created (typically in the GUI main figure window, a handle to which is provided as argument when this function is called by the GUI class).

11.4 Update Visualization

The function `update` is used to update what is displayed in the visualization, such that it reflects the current state of the simulation. This typically means to fetch the specified element components from the simulator and update all plots. The function is called by the GUI in each step.

Part IV

Tutorials and Examples

12 Using the Graphical User Interface

This tutorial gives an overview on how the Graphical User Interface (GUI) in COSIVINA works and how you can use it to test and adjust a neurodynamic model. The descriptions are geared primarily towards the example simulations provided with the framework (in the folder `examples`), but all interfaces that use the `StandardGUI` class show the same general behavior (although the controls provided in the GUI may vary). An overview over the available simulators can be found in the `README` file in the `examples` folder.

The example simulations are started by executing the launcher files in the Matlab command window (e.g. `launcherOneLayerField.m`). Each launcher file sets up an architecture and an accompanying GUI, and then starts that GUI. This is done with the command

```
gui.run();
```

at the end of each launcher file.

This initializes the GUI, iterates the steps of the associated `Simulator` object, updates the visualizations and manages all interface operations. When the GUI window opens, the simulation immediately and continuously runs: Simulation steps are performed and all elements are continuously updated. This may not be obvious if the model is in a stable state, but it is nonetheless taking place.

The simulators all show one or more visualizations of the current state of the neural fields in the model, and provide a number of sliders that control certain parameters of elements in the simulation, as well as a set of global control buttons. One-dimensional fields are typically visualized as plots over the feature space. The default color code is blue for field activation, red for field output (the sigmoid of the activation), and green for inputs to a field. The input plot often incorporates the field resting level, such that it is aligned with the field activation in the absence of interaction effects. The output plot is often scaled up to make it more visible. For fields over a two-dimensional feature space, the activation distributions (and sometimes also the field outputs) are shown as color-coded images or surface plots.

You can now affect model parameters by using the sliders (click on arrows for changes in small steps, click on the slider bars for larger steps, or move the slider directly for continuous changes). The label next to each slider indicates what parameter is controlled by it and gives the current value of that parameter. Hovering with the mouse over a slider or other control will generally show a more detailed description of what that control does. In the basic example simulators (such as `launcherOneLayerField.m`), sliders for the field and interaction parameters are arranged in the top part of the controls grid, sliders for the stimulus parameters in the bottom part. Here, you can activate a stimulus by setting the stimulus strength via the sliders on the bottom right, then change its width and position with the left and center sliders. The field activation should then follow the stimuli.

Most simulations offer the same set of default buttons to globally control the simulation. The Pause button simply pauses the simulation, that is, it prevents simulation steps from being performed and simulation time from being incremented. All controls remain usable while the simulation is paused, and parameter changes still take effect. However, these effects may only become visible when the simulation is resumed. If you want to keep a simulation open in the background but are not currently using it, it may be a good idea to pause it in order to reduce processing load.

The Reset button resets the state of the simulation by re-initializing all elements in the simulator. It does not reset or otherwise affect the parameters of the elements, just their state (e.g. the activation patterns in the neural fields are set back to the resting level, and memory traces are set back to zero). It also sets the simulation time back to the value t_0 specified in the `Simulator` object (zero by default). This means that if you have time-dependent stimuli defined for your simulation, their sequence will start over.

The Parameters button will open the parameter panel for the running simulation in a separate window. This panel shows a drop-down menu, by default containing a list of all elements in the simulator (although this can be adjusted when defining the GUI). For the selected element, the panel then lists all of its parameter values. Fixed parameters are grayed out; for non-scalar parameters, only their type may be shown, not the actual values. All other parameters values may be changed by setting their value in the edit field, then pressing the Apply button at the bottom of the panel. Parameter changes that have not been applied are not retained when you select another element from the drop-down menu. The parameter panel can be closed by pressing the Parameters button again.

With the Save button, it is possible to save the complete parameter set of the currently running simulation in a configuration file in JSON format. Pressing the button will open a file dialog where you can choose a file location. The simulation continues after the file has been saved. The configuration file lists all elements in the simulator with their connections and parameters in a human-readable format. It does not save the current state of the simulation (like activation distributions in fields). At a later time, you can then load from such a parameter file using the Load button (which again opens a file dialog). When you load from a parameter file, the whole simulation is re-initialized and its previous state is lost. Parameter files are intended mainly to store successful parameter sets, but you may also edit the values in the file and then load it again. You may even change the elements and connectivity in the JSON file, as long as it remains compatible to the GUI. The GUI will warn you about missing elements, but still load parameters for all matching elements (determined by class and label) from it. When working with multiple configuration files, the function `compareConfigFiles` (in the `auxiliary` folder) can be used to determine what elements or parameters have been changed between them.

The Quit button ends the simulation and closes the GUI window. The last state of the simulation is still retained in the `Simulator` object (the variable `sim` in the example launchers). Note that this is not guaranteed if you terminate the GUI by other means than the Quit button, though it will typically still be the case. This means you may access elements of that simulator, read out their components, manually change parameters, or create a copy of the simulation (see Section 14 and example below). To continue the simulation in the GUI, execute `gui.run()` again (if you execute the launcher script again, a new simulator will be created and the previous state will be lost).

The `run` function of the GUI may be called with additional arguments: A maximum simulation time, a flag to force initialization, a flag to close the simulator when the GUI is closed, and a simulator handle. For instance, the call `gui.run(1000, true);` runs the simulation and quits when simulation time reaches 1000; also, it forces an initialization of the simulator when the GUI is started (so any previous state is lost). The fourth argument (specifying a new simulator handle) can be used like this:

```
gui.run(); % run sim in the GUI, quit at some point
sim2 = sim.copy(); % create a copy of sim
gui.run(inf, false, false, sim2); % try something in the copy
gui.run(inf, false, false, sim); % continue where the copy was made
```

Here, the GUI is first run with the original `Simulator` object `sim` unto some point, and

then is closed with the Quit button. A copy `sim2` of the current simulation is created. The GUI is then launched again with this copy, continuing at the same point in the simulation where it had just been interrupted. For example, you can try out one possible action in the simulation after you have build up a certain activation pattern in the fields. The GUI is closed again with the Quit button, and then restarted with the original `Simulator` object. This object is still in the same state as it was when the copy was made, so you can continue from this state again and try out another course of action. In the same fashion, you could also create more copies to try out different things, always starting from the same state.

13 Building a Simple DNF Architecture

In this section we illustrate how a basic DNF architecture can be built in a Matlab script. We will then show how to use that architecture in the following sections. You find the complete code for this example in the COSIVINA folder under `examples/exampleA.m`.

(Note: It is in principle also possible to create a full architecture by directly editing a parameter file in JSON format, and then loading the settings from that file. If you intend to use this approach, we suggest that you study the parameter file of an existing architecture. The JSON format is readable and the storage of the elements in the file largely self-explanatory, but strict adherence to this format is required to be able to load it from Matlab.)

As a first step, we create an empty `Simulator` object by calling the class constructor. The default settings will be adequate in most cases, so the object (called `sim` throughout the example) can be created by calling

```
sim = Simulator();
```

When we show the properties of the object (by typing its name in the Matlab command line), we will see something like this:

```
sim =
  Simulator handle
  Properties:
        nElements: 0
         elements: {}
    elementLabels: {}
      initialized: 0
           deltaT: 1
            tZero: 0
                t: 0
```

The object does not contain any elements and is not initialized.

We can now successively add elements to the `Simulator` object. We will begin with a one-dimensional neural field as first element:

```
sim.addElement(NeuralField('field u', 100, 10, -5, 4));
```

The `addElement` method of the `Simulator` class is used to add the new element to the empty architecture. The call of the constructor `NeuralField` returns a handle to a newly created element with the given parameters. When you want to add an element and are unsure about the required parameters and their order in the constructor call, you can use the command window help for all elements (as well as controls and visualizations), for instance:

```
>> help NeuralField
  NeuralField (COSIVINA toolbox)
    Creates a dynamic neural field (or set of discrete dynamic nodes) of
    arbitrary dimensionality with sigmoid (logistic) output function. The
    field activation is updated according to the Amari equation.

  Constructor call:
  NeuralField(label, size, tau, h, beta)
    label - element label
    size - field size
    tau - time constant (default = 10)
    h - resting level (default = -5)
    beta - steepness of sigmoid output function (default = 4)
```

In our example, we give this element the label `field u`, by which it can later be referenced to set up connections or create visualizations of its components. The next argument to the `NeuralField` constructor specifies its size. The scalar argument `100` is interpreted as specifying a one-dimensional field (which is always stored as a row vector). We could also have given the argument as `[1, 100]` to obtain the same result. The other arguments of the constructor specify further parameters of the field (see the specifications of the this element class above). Some or all of these may be omitted if they match the default values (as they do here) and the actual values are to be set e.g. via the GUI.

Note that we can also perform the above operations in two separate steps:

```
hFieldU = NeuralField('field u', 100, 10, -5, 4);
sim.addElement(hFieldU);
```

Here, we first called the constructor method and obtained an explicit handle `hFieldU`, and then added the element to the simulator via this handle. The handle can then later be used to access the element's properties (but a handle can also be obtained at any time from the `Simulator` object through the `getElement` method). We will in the following examples generally use the more compact form of the function call shown above.

The neural field we have added does not have any lateral interactions, we have to add these as a separate element. Different classes can be used to generate lateral interactions for a one-dimensional field, such as `GaussKernel1D` or `MexicanHatKernel1D` (a difference of two Gaussian kernels). We will use the class `LateralInteractions1D` here, which creates a Mexican-hat kernel with an additional global component:

```
sim.addElement( ...
  LateralInteractions1D('u -> u', 100, 4, 15, 10, 15, 0), ...
  'field u', 'output', 'field u', 'output');
```

Again, we have created the element itself by calling its constructor. We give the new element the descriptive label `u -> u`. Since this element is to be connected to `field u`, their sizes must match (several element types exist to couple elements of different sizes or dimensionality if necessary). This is specified in the second argument to the constructor. The following arguments determine the interaction strengths and widths. The element handle returned from the constructor is the first argument to the function `addElement`. Here, additional arguments are added to set up the connectivity of the new element. These arguments specify, in the order they are given: The new element should receive input from `field u` (2nd argument), namely the component `output` of this element (3rd); and it should itself

project back to the element `field u` (4th), which receives the component `output` of the new element as input (5th).

Note that the arguments specifying the connectivity are all character strings. Existing elements in the architecture can be addressed via their label (this is why a unique label must be given to every element in an architecture). Components are addressed via their name (as a character string). This matches the name of the property in the element object (for instance, every element of class `NeuralField` has a property `output`).

Since `output` is the name of the default output component of both the `NeuralField` and the `LateralInteractions1D` classes (as well as many others), we can simplify the above call to one of these forms:

```
sim.addElement( ...
  LateralInteractions1D('u -> u', 100, 4, 15, 10, 15, 0), ...
  'field u', [], 'field u', []);
% or
sim.addElement( ...
  LateralInteractions1D('u -> u', 100, 4, 15, 10, 15, 0), ...
  'field u', [], 'field u');
```

We now add two Gaussian stimuli to the architecture:

```
sim.addElement(GaussStimulus1D('stim A', 100, 5, 6, 25), ...
  [], [], 'field u');
sim.addElement(GaussStimulus1D('stim B', 100, 5, 8, 75), ...
  [], [], 'field u');
```

Stimuli do not receive any inputs, therefore the second and third arguments remain empty. Both stimuli should project to the existing element `field u` in the architecture, so that is the fourth argument. One could make it explicit that `field u` should receive the component `output` of the two stimuli as input, but since this is the default anyway, we omit the optional 5th argument. Note that we have to create two separate objects of the GaussStimulus1D class to add them to the simulator object. It would not be possible, for instance, to create one object of the GaussStimulus1D class and obtain the handle for it outside of the `addElement` function call, and then call the `addElement` function twice with this same object handle.

These are all elements we will use in the first architecture. When we show the properties of the object `sim` again, we will see the following:

```
sim =
  Simulator handle
  Properties:
        nElements: 4
         elements: {[1x1 NeuralField]  [1x1 LateralInteractions1D]
                    [1x1 GaussStimulus1D]  [1x1 GaussStimulus1D]}
    elementLabels: {'field u'  'u -> u'  'stim A'  'stim B'}
      initialized: 0
           deltaT: 1
            tZero: 0
                t: 0
```

Four elements now exist, but the object is still not initialized. We can now either create a GUI for this simulator (see Section 15), or operate it in an offline mode without handing over control to a GUI (Section 14 and 16).

14 Running a Simulation in Offline Mode

We will continue here to work with the architecture set up in the previous section, the full code for this tutorial is also contained in the file `exampleA.m`. The simplest way to run the simulation without a GUI is to call the `run` method of the `Simulator` class. We will describe this below, but first, we will show how to perform the required operations individually. First, the simulator object has to be initialized. If we have set up a new architecture, it may be helpful to use the `tryInit` method, which will give us detailed information if anything goes wrong during initialization:

```
sim.tryInit();
```

This should run through without any problems in our example, and the simulator object is now initialized. We can continue to check our architecture by performing a single simulation step with the `tryStep()` function:

```
sim.tryStep();
```

If there are any problems in the architecture – such as size mismatches between connected elements – this function will throw an error and inform us about which element caused the problem. It also performs an actual step of the simulation. It is mostly sufficient to perform just one of these trial steps, since most errors should appear immediately when running the simulation. Again, there should be no problems in the example we give here.

If we have used the architecture before (or are confident that everything is correct), we can omit the previous steps and proceed directly to the actual simulation. Still, we have to initialize the simulator object first:

```
sim.init();
```

All elements are now initialized, and all the components have been created as matrices of the correct size. Some of these are still filled with zeros (e.g. in the connective elements), some already have meaningful content (true for the dynamic elements and stimuli). For instance, we can plot one of the Gaussian stimuli in the architecture:

```
plot(sim.getComponent('stim A', 'output'));
```

We used the `getComponent` method here to directly access the component `output` of the element labeled `stim A`, and should obtain the plot of a Gaussian curve. In the same way, we can show the activation of the neural field:

```
plot(sim.getComponent('field u', 'activation'));
```

Currently, the newly initialized field is at its resting level everywhere. It will change under the influence of the stimuli when we run the simulation.

We can go through simulation steps manually using the `step` function, for instance in this form:

```
for i = 1 : 10
  sim.step();
end
```

If we plot the neural field activation again in the same way as before, we will see how its activation has increased locally around the centers of the two stimuli. The steps also counted up the internal timer of the simulator object:

```
>> sim.t
ans =
    10
```

If we perform another 10 steps and repeat the plot of the field activation, we can see that supra-threshold activation peaks have formed and observe the effect of the lateral interactions in the form of depressed activation around the peaks. For formal analysis, we can also store these activation patterns at different times during the simulation.

In addition to plotting or storing the state of the system during the simulation, we can also change the properties of elements. For instance, we can now turn off one of the stimuli by setting its `amplitude` parameter to zero. The easiest (and recommended) way of doing this is to use the `setElementParameters` function of the `Simulator` class:

```
sim.setElementParameters('stim B', 'amplitude', 0);
```

We can plot the output of the stimulus again to confirm that it is now flat, and can then continue to run the simulation with further calls of the step function.

It is also possible to access elements and their parameters more directly. For instance, we can achieve the same parameter change with the following code:

```
hStimB = sim.getElement('stim B');
hStimB.amplitude = 0;
hStimB.init();
```

Here, we first obtain a handle of the element we want to change, then access the parameter (a property of the element object). After changing the parameter, the element has to be re-initialized for the change to take effect (the stimulus pattern in the `GaussStimulus1D` element is only created during initialization). The `setElementParameters` function takes care of this initialization (and sometimes an additional call of the `step` function) for us whenever necessary. You have to use the manual access however if you want to change certain parameters that are classified as Fixed.

Finally, for this scenario it is also possible to directly use a time-dependent stimulus, which is provided by the element `TimeGaussianStimulus1D`. This stimulus can be set up beforehand to be turned off at a certain time, and would not require any parameter changes during the simulation. An example for this is described in Section 16.

We can simplify the code for performing the simulation by utilizing the `run` method of the `Simulator` class. The operations described above (from the initialization on) can then be replaced with the following piece of code:

```
sim.run(10, true);
sim.setElementParameters('stim B', 'amplitude', 0);
sim.run(20, false);
```

The first call of `run` initializes the simulator (explicitly requested by setting the second argument to `true`) and runs it until it reaches simulation time `t = 10`. In this case, this is equivalent to the ten steps that we explicitly performed in a loop above. Note, however, that it could be a different number of steps depending on the simulator's `tZero` and `deltaT` parameters. The parameter `tZero` determines the value to which the simulation time is set during initialization. The parameter `deltaT` controls the temporal sampling: For instance, if we set `deltaT = 0.1`, the call `sim.run(10, true)` will perform one hundred steps, but each will only create a smaller change in the field activation. The final state of the system will be qualitatively the same, with some numerical differences.

In the second call of run in the piece of code above, we specify that the simulation should now run until it reaches simulation time 20. The second argument false indicates that the simulation should not be re-initialized but continue from its previous state. We could also omit this second argument here, since the run command does by default not perform an initialization if the simulator is already initialized.

Finally, if you have a simulation that is connected to external programs or hardware, you may need to close this connection when the simulation is finished. You can do this by calling

```
sim.close();
```

or by using the optional third argument of the run function. Both will call the close function of all elements in the simulator. In our example, this would have no effect, since these functions are all empty.

15 Creating a Graphical User Interface

The GUIs in the COSIVINA framework are created for a specific architecture, and linked to a specific Simulator object. We will in this example create a simple GUI for the small architecture described in the previous sections. To do so, we first create a StandardGUI element, with general GUI settings specified in the constructor. Then we add a visualization of the field activation and several control elements to the GUI. You can find the complete code in the file exampleB.m.

15.1 Creating the GUI Object

The StandardGUI object is created via a constructor call, with arguments specifying global parameters of the GUI. We assume that a Simulator object with elements as described in Example A exists in the workspace.

```
gui = StandardGUI(sim, [50, 50, 700, 500], 0.05, ...
  [0.0, 1/3, 1.0, 2/3], [1, 1], 0.1, ...
  [0.0, 0.0, 1.0, 1/3], [5, 3]);
```

The first argument sim is a handle to the Simulator object that is connected to this GUI. The second argument specifies the figure size and position for the GUI main window on start up (it can be freely resized while it is running). The third argument specifies the duration of the pause that is introduced after every simulation step. The value used here is reasonable for relatively small architectures, where the pause makes it simpler to follow the evolution of the field activation. For larger architectures and field sizes, this value should be set to zero, since the computation time slows down the simulation sufficiently for viewing it (or even more than that).

The arguments in the next line specify the set-up of the visualization grid of the GUI (see also Figure 4.1): We want it to occupy the upper two-thirds of the window, and contain only a single grid cell (for a single visualization). The last argument in this line specifies a padding around the visualization. The arguments in the last line analogously specify the settings for the controls grid: In this example, the grid should cover the bottom third of the GUI main window, with 5×3 cells for individual control elements. No padding is provided for the control elements. There are two more optional arguments for the constructor call which allow customization of the parameter panel, but we do not use them in this example.

GUI parameters may be changed after the constructor call. For instance, if we find the pause duration to be inadequate, we can change the behavior of an existing GUI in the following form:

```
gui.pauseDuration = 0.025;
```

Note that changes to the visualization or control grid settings do not affect graphical elements that have already been added.

15.2 Adding Visualizations

The visualizations display one or more element components and are typically updated after each simulation step. In this example, we include only a single visualization, a plot of the field activation, output, and stimuli, using the `MultiPlot` visualization class. Creating a customized visualization requires the specification of many parameters, so the constructor calls can become a bit cluttered. Use the Matlab help for the visualizations, e.g.

```
>> help MultiPlot
```

to see instructions for defining each visualizations and examples of its use.

We add the visualization with the following command:

```
gui.addVisualization(MultiPlot(...
  {'field u', 'field u', 'stim A', 'stim B'}, ...
  {'activation', 'output', 'output', 'output'}, ...
  [1, 10, 1, 1], 'horizontal', ...
  {'YLim', [-10, 10], 'Box', 'on'}, ...
  {{'b', 'LineWidth', 2}, {'r'}, {'g'}, {'g'}}, ...
  'field u', 'field position', 'activation/ouput/input'), ...
  [1, 1]);
```

The outer method call `gui.addVisualization(...)` is the generic function for adding a visualization element to the GUI, with a constructor call for the `MultiPlot` object yielding the first argument. This constructor call takes several arguments to fully specify the plots and the axes setup. The first two arguments are cell arrays of strings that together specify *what* is plotted: Each pair of entries from these cells specifies the source for one plot, with the first cell array containing the element labels, the second the component names. Here, we want to plot: The activation of `field u`, the output of `field u`, and the outputs of both `stim A` and `stim B`. The third argument of the constructor specifies the scales for these plots. We scale up the field output tenfold to make changes in this component more visible (since it only ranges from zero to one). The next argument specifies that the plot should be oriented horizontally.

Next, we can specify settings for the axes. These are the same axes properties that can also be provided in the `axes` call in Matlab, given as a list of property/value pairs in a cell array. In this case, we set the limits of the y-axis to the range `[-10, 10]`, and specify that the axes should be enclosed by a box. In a similar way, we can set the properties for each plot in the following argument. Here, we use a cell array of cell arrays, with one inner cell array for each individual plot. Within each inner cell array, we can specify line type and color with a single string (as documented in the Matlab `plot` function), and/or list property/value pairs for the plot. In this example, we want the line for the field activation (the first plot) to be blue and a bit thicker, the other plots to be in red and green, respectively. Finally, in the last three arguments to the constructor call, we specify axes labels and a title for the visualization.

There is then one more argument to the outer function: The vector [1, 1] specifies the position of this visualization within the grid. We could also provide another argument here specifying a size of the visualization in the grid. For instance, the GUI in the file `launcherCoupling.m` uses a 4×4 visualizations grid, in which two plots of one-dimensional fields are placed (one horizontal, one vertical), as well as an image for a two-dimensional field. The arrangement of these visualizations, shown in Figure 15.2, is created as follow:

```
gui.addVisualization(MultiPlot(...), [4, 2], [1, 3]);
gui.addVisualization(MultiPlot(...), [1, 1], [3, 1]);
gui.addVisualization(ScaledImage(...), [1, 2], [3, 3]);
```

If one does not want to use the visualizations grid, it is always possible to add an explicit position argument directly to the constructor call of the visualization element instead. The position arguments in the call of the `addVisualization` method should then be omitted.

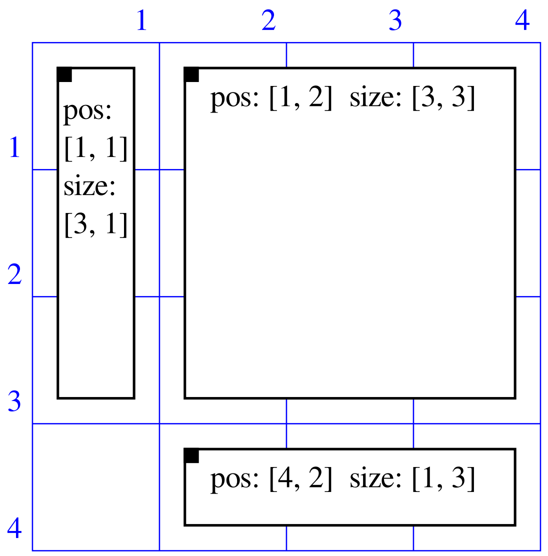

Figure 2: Arrangement plots (for coupled one-dimensional and two-dimensional fields) in a 4×4 visualizations grid.

Since the `MultiPlot` constructor call can become so cluttered if a large number of plots is created, an alternative method is provided to add plots successively. The call above can be replaced by the following operations:

```
hMP = MultiPlot([], [], [], 'horizontal', ...
  {'YLim', [-10, 10], 'Box', 'on'}, {}, ...
  'field u', 'field position', 'activation/ouput/input');
hMP.addPlot('field u', 'activation', 1, {'b', 'LineWidth', 2});
hMP.addPlot('field u', 'output', 10, {'r'});
hMP.addPlot('stim A', 'output', 1, {'g'});
hMP.addPlot('stim B', 'output', 1, {'g'});
gui.addVisualization(hMP, [1, 1]);
```

Here, the arguments in the constructor call specifying the individual plots and their sources remain empty, and the plots are then added individually. Note that the visualization element may only be added to a GUI once it is complete.

15.3 Adding Controls to Change Element Parameters

For tuning the model, it is often convenient to change parameters while the simulation is running and directly observe the effects. One way to do that is via the parameter panel, which allows access to the parameters of all elements. For settings that are likely to be changed frequently during the online simulation, an even more direct way to access them is desirable. To this end, graphical control elements can be added to the GUI. These controls can be arranged in a control grid analogously to the visualizations grid.

We can add a slider to control the resting level of the neural field in the example architectures using the `addControl` method of the `StandardGUI` class and the controls class `ParameterSlider`:

```
gui.addControl(ParameterSlider('h', 'field u', 'h', [-10, 0],...
  '%0.1f', 1, 'resting level of field u'), ...
  [1, 1]);
```

The constructor of the `ParameterSlider` class takes as first argument a descriptive label that is displayed next to the slider. The label can be chosen freely, and does not need to match any class or parameter name (it should however be relatively short). The second argument is the label of the element accessed by this slider, and the third one the name of the controlled parameter in the element. Here, we want to control the parameter `h` (the resting level) of the element labeled `field u`. The element label must exist in the `Simulator` object linked to the GUI, and the element class must have a parameter of the specified name. The following arguments specify slider range, number format for the display of the parameter value, scaling factor and tool tip (see Controls Reference for details).

We can add sliders for the lateral interaction strengths in the same way. These controls are all connected to different parameters of the element `u -> u`:

```
gui.addControl(ParameterSlider('c_exc', 'u -> u', 'amplitudeExc', ...
  [0, 40], '%0.1f', 1, 'strength of lateral excitation'), [2, 1]);
gui.addControl(ParameterSlider('c_inh', 'u -> u', 'amplitudeInh', ...
  [0, 40], '%0.1f', 1, 'strength of lateral inhibition'), [2, 2]);
gui.addControl(ParameterSlider('c_gi', 'u -> u', 'amplitudeGlobal', ...
  [0, 1], '%0.1f', -1, 'strength of global inhibition'), [3, 1]);
```

The sliders are placed in consecutive locations in the grid via the second argument of the `addControl` method. Note that in the last line, we created a global inhibition slider (labeled `c_gi`) with a scaling factor of -1. This means that when we move that slider to the right (to more positive values), the controlled parameter `amplitudeGlobal` becomes more negative.

We furthermore add controls for the stimulus settings, with each one slider controlling stimulus position and one controlling stimulus strength for each of the elements `stim A` and `stim B`:

```
gui.addControl(ParameterSlider('p_s1', 'stim A', 'position', ...
  [0, 100], '%0.1f', 1, 'position of stimulus 1'), [4, 1]);
gui.addControl(ParameterSlider('c_s1', 'stim A', 'amplitude', ...
  [0, 20], '%0.1f', 1, 'stength of stimulus 1'), [4, 2]);
```

```
gui.addControl(ParameterSlider('p_s2', 'stim B', 'position', ...
  [0, 100], '%0.1f', 1, 'position of stimulus 2'), [5, 1]);
gui.addControl(ParameterSlider('c_s2', 'stim B', 'amplitude', ...
  [0, 20], '%0.1f', 1, 'stength of stimulus 2'), [5, 2]);
```

For the position sliders, we adjusted the slider range to the size of field u, such that the stimuli can be centered at any position in the field.

A single slider may also control multiple parameters. For instance, we can set up a slider to adjust both stimulus strengths simultaneously with the following command:

```
gui.addControl(ParameterSlider('c_s', {'stim A', 'stim B'}, ...
  {'amplitude', 'amplitude'}, [0, 20], '%0.1f', 1, ...
  'stength of both stimuli'), [6, 1]);
```

The controlled elements and parameters are here given as cell arrays of strings, with each pair of corresponding strings from the two cell arrays fully specifying one parameter controlled by the slider. We may also want to simply turn stimuli on and off (with a specific strength for the on state). In this case, we can use the `ParameterSwitchButton`. Here, we have to specify the desired values for the controlled parameters for the pressed on non-pressed state of the button:

```
gui.addControl(ParameterSwitchButton('stimuli on', ...
  {'stim A', 'stim B'}, {'amplitude', 'amplitude'}, ...
  [0, 0], [6, 6]), [6, 2]);
```

If the last two control elements are implemented in addition to the individual sliders for the stimulus strength, there is a possibility of conflicts between the controls: For instance, if the stimulus strengths are set to different values by the individual sliders, the slider position of the combined slider cannot reflect this. In the framework, this will not lead to errors in the simulation, but there can be inconsistencies between displayed values on the controls and the actual parameter values. The behavior of the GUI is as follows: The last control element that is activated (button clicked, slider moved, etc.) sets all associated parameters to values specified by this control. Other control elements may be updated to reflect that change if possible (true e.g. for sliders), or they remain unchanged (true e.g. for buttons). Similar situations can also occur if parameters are changed in the parameter panel.

15.4 Adding Global Controls

Special control elements to globally control the simulation can be added in the same way as the controls to change element parameters. The `StandardGUI` supports the following control mechanism:

- pause the simulation
- reset the simulation (by re-initializing all elements and resetting the simulation time)
- open and close the parameter panel
- save parameters to file
- load parameters from file
- quit the simulation

These functions can be performed by setting control flags in the StandardGUI object via a GlobalControlButton. The controls for these standard functions can be created in the GUI as follows:

```
gui.addControl(GlobalControlButton('Pause', gui, ...
  'pauseSimulation', true, false, false, 'pause simulation'), ...
  [1, 3]);
gui.addControl(GlobalControlButton('Reset', gui, ...
  'resetSimulation', true, false, true, 'reset simulation'), ...
  [2, 3]);
gui.addControl(GlobalControlButton('Parameters', gui, ...
  'paramPanelRequest', true, false, false, 'open parameter panel'), ...
 [3, 3]);
gui.addControl(GlobalControlButton('Save', gui, ...
  'saveParameters', true, false, true, 'save parameter settings'), ...
  [4, 3]);
gui.addControl(GlobalControlButton('Load', gui, ...
  'loadParameters', true, false, true, 'load parameter settings'), ...
  [5, 3]);
gui.addControl(GlobalControlButton('Quit', gui, ...
  'quitSimulation', true, false, false, 'quit simulation'), ...
  [6, 3]);
```

The constructor calls for these buttons are generally independent of the type of the GUI and the simulation, and can just be copied and rearranged in the desired fashion for all GUIs.

An additional form of global control element is implemented in the PresetSelector class. This control creates a drop-down menu from which one of several prepared parameter settings can be selected. It invokes the same operation as the Load button used above, but instead of opening a file selection dialog uses one of a set of predefined files. An example for its use can be seen in the file launcherField1l_preset.

You can now run the simulation in this GUI by calling gui.run(). See Section 12 on GUI usage and additional options in starting the GUI. Alternatively, it is also possible to use a GUI with additional custom code, or to use it only for visualization. This is described in the following section.

16 Using the GUI in Customized Simulations

In some cases, you may want to have a visualization of your simulation as it is provided by the GUI, but not actually hand over control to this GUI. For instance, you may want to run many trials with a fixed time course, and observe how the model behaves. Or, you may want to have custom code for analysis or modification of the model behavior integrated into the simulation, in a way analogous to what was described in Section 14. This section describes how to achieve this.

The first scenario uses the GUI only for visualization, and all control elements are omitted. The code for this scenario can be found in the file exampleC. An example architecture is set up similar to the one used before. However, here we will use time-dependent stimuli to set up a simulation time course without the need to change parameter values while the simulation is running. The architecture is created with the following code:

```
% create object sim by constructor call
```

```
sim = Simulator();

% shared element parameters
fieldSize = 100;

% timing parameters
tMax = 200;
tStimOn = 25;
tBoost = 100;

% add elements
sim.addElement(NeuralField('field u', fieldSize, 10, -5, 4));
sim.addElement(...
  LateralInteractions1D('u -> u', fieldSize, 4, 15, 10, 0, -0.6), ...
  'field u', 'output', 'field u', 'output');
sim.addElement(...
  TimedGaussStimulus1D('stim A', fieldSize, 5, 4, 25, [tStimOn, inf]), ...
  [], [], 'field u');
sim.addElement(...
  TimedGaussStimulus1D('stim B', fieldSize, 5, 4, 75, [tStimOn, inf]), ...
  [], [], 'field u');
sim.addElement(TimedCustomStimulus('boost', 2, [tBoost, inf]), ...
  [], [], 'field u');
sim.addElement(NormalNoise('noise', fieldSize, 0.5), [], [], 'field u');
```

We have first specified some shared parameter and timing values that are used in multiple elements. (It is good practice to define these as explicit variables, so they can be changed in one place.) The architecture consists of a neural field with lateral interactions, just as in the previous examples. The Gaussian stimuli are now replaced by time-dependent Gaussian stimuli. The constructors for these take one additional argument, an $N \times 2$ matrix of start and stop times. Here, the stimuli are only activated for one time period, so the matrix has only one row. Also note that we have set the end time to infinite, so the stimuli will remain active as long as the simulation is runnig.

Beside these localized inputs, we have defined a global boost input, implemented through the element `TimedCustomStimulus`. In the constructor call, the stimulus pattern is simply set to 2, so this value will be added to every point in the field. We could also specify a pattern here that is the same size as the field itself, but we just want a homogeneous input. Furthermore, a noise input is added to the field, so that the simulation will produce stochastic results.

For the most basic visualization, we set up a GUI with a plot of the field and the Gaussian inputs:

```
% create the gui object
gui = StandardGUI(sim, [50, 50, 700, 500], 0.05, ...
  [0.0, 1/4, 1.0, 3/4], [1, 1], 0.1, ...
  [0.0, 0.0, 1.0, 1/4], [3, 3]);

% add a plot of field u (with activation, output, and inputs)
gui.addVisualization(...
  MultiPlot({'field u', 'field u', 'stim A', 'stim B'}, ...
```

```
  {'activation', 'output', 'output', 'output'}, [1, 10, 1, 1], ...
  'horizontal', {'YLim', [-12.5, 12.5], 'Box', 'on'}, ...
  {{'b', 'LineWidth', 2}, {'r'}, {'g'}, {'g'}}, ...
  'field u', 'field position', 'activation/ouput/input'), ...
  [1, 1]);
```

As a new visualization element, we also add a display of the simulation time to this GUI:

```
gui.addVisualization(TimeDisplay(), [1, 3], [1, 1], 'control');
```

This text-based visualization is placed in the control grid of the GUI here, by giving the string `'control'` as optional last argument.

We can now run the simulation manually, by calling the step function in a loop, and showing the state of the simulation in the GUI for each step. In the simplest form, the code looks like this:

```
sim.init();
gui.init();

while sim.t < tMax
  sim.step();
  gui.updateVisualizations();
  pause(0.05);
end
```

First both the simulator and the GUI are initialized. A loop is set up with the simulation time used as termination condition (the time `sim.t` is automatically incremented by `deltaT` in every call of the step function). In each iteration, a simulation step is performed and the current state of the simulation is shown in the GUI by calling `gui.updateVisualizations`. A pause may be introduced to make it easier to follow the simulation. The GUI may optionally be closed at the end of the simulation by adding the function call `gui.close();` after the while-loop.

We can also add additional custom code into this loop to change the behavior of the simulation or to analyze the results. For instance, we may stop the simulation as soon as a sufficiently strong activation peak has formed in the field, and then determine the locations of all peaks in the field. The extended while-loop then looks like this:

```
while sim.t < tMax
  sim.step();
  gui.updateVisualizations();
  pause(0.05);

  act = sim.getComponent('field u', 'activation');
  if any(act > 5)
    [nPeaks, peakPos] = singleLinkageClustering(act > 0, 3, 'circular');
    break;
  end
end
```

Here, the method `sim.getComponent` is used to read out the field activation in every simulation step. If the activation at any point exceeds a threshold, the locations of all activation

peaks in the field are determined using the function `singleLinkageClustering` (an auxiliary function included in COSIVINA). The while-loop is then interrupted. If no peak forms, the while-loop still ends at time `tMax`.

Rather than visualizing each step, it is also possible to show the state of the simulation only at specific time steps. In the example above, this could be done by introducing an if-clause and only calling the GUI update at intervals or at predefined times. In the following example, the complete simulation is run (repeatedly for multiple trials), and only the result is shown (paused until a key is pressed). To this end, we create a loop over trials, and replace the call of `sim.step()` with a call to `sim.run(tMax, true)`, which initializes the simulation and runs it until the specified time is reached.

```
sim.init();
gui.init();

nTrials = 5;
for i = 1 : nTrials
  sim.run(tMax, true);
  gui.updateVisualizations();
  pause;
end
gui.close();
```

The second scenario described in this section uses the full GUI with all of its control elements, but inserts some custom code into each simulation step. The code for this scenario is given in the file `exampleD`. The example uses the same simple architecture and the same GUI as introduced in `exampleB`.

Inserting custom code in the simulation is not possible when using the method `gui.run`. So instead, we use a while-loop over time steps again, and call the method `gui.step` within this loop. In the simplest form, the code to run the GUI in this fashion looks like this:

```
sim.init();
gui.init();

while ~gui.quitSimulation && sim.t < tMax
  gui.step();
end
```

Again, we first have to initialize the simulation and the GUI. The while-loop is terminated either when a specified time `tMax` is reached in the simulation, or if the GUI is closed. The latter is determined via the boolean property `quitSimulation` of the GUI object, which is set to true if either the `Quit` button is pressed, or if the GUI figure window is closed. Note that the GUI object is still present in the workspace and still valid after the figure window has been closed.

The `step` method of the GUI object covers all operations that are also performed in each time step when using the `gui.run` method. It calls the step function of the connected simulator object (to update field activations etc.), updates all visualizations, checks for any changes in the control elements and applies these changes, and finally updates the control elements themselves if necessary. It also introduces a brief pause for viewing the simulation (as specified by the GUI object's `pauseDuration` property), and it deals with presses of the control buttons (e.g. for pause, save and quit).

Note that such control button presses can influence the number of iterations in the while-loop. While the `Pause` button is pressed, the simulator object is not updated and

the time variable `sim.t` is not incremented. (If you want to check within the code whether the simulation is paused, access the boolean property `pauseSimulation` of the GUI object.) And when the `Reset` button is pressed, the timer is set back to its initial value.

We can now add custom code into the while loop that accesses and changes the status of the simulator object (or of the GUI). For instance, we may want to turn off the input as soon as an activation peak of sufficient strength has formed in the field. The extended while-loop for this behavior looks like this:

```
while ~gui.quitSimulation && sim.t < tMax
  gui.step();

  if any(sim.getComponent('field u', 'activation') > 5)
    sim.setElementParameters('stim A', 'amplitude', 0);
    sim.setElementParameters('stim B', 'amplitude', 0);
    gui.checkAndUpdateControls();
  end
end
```

We access the field activation in the same way as in the previous example. Then we change the amplitudes of the stimuli to zero using the `setElementParameters` method (internally, this will cause a re-initialization of the two stimulus elements to apply this change). Finally, we call the method `gui.checkAndUpdateControls`. This is necessary here to make sure that the sliders in the GUI correctly reflect the externally induced change in stimulus amplitudes.

In this fashion, it is possible to execute any custom code to access and change the state of the simulation, or to analyze its behavior. Keep in mind that while this approach may be the easiest way to generate a certain effect in the simulation, it is not necessarily the most elegant one. If the desired effect should form a part of the modeled behavior, then it should ideally be integrated into the neurodynamic architecture, rather than being performed algorithmically outside of the system. And if the effect cannot be achieved with the existing elements in the COSIVINA framework, you may consider creating a new element, as described in Part III.

17 Building Architectures with Two-Dimensional Fields

In this example we will describe another simple architecture, now including two-dimensional fields and coupling between fields of different dimensionality. We will describe in detail how lateral interactions are set up in two-dimensional fields and how projections between one-dimensional and two-dimensional fields can be implemented. The complete code can be found in the file `exampleE.m`. Additional examples of combining fields of different dimensionality can be found in the files `launcherCoupling`, `launcherCoupling3D`, and `launcherTransformation`.

17.1 Fields, Inputs, and Lateral Interactions

We create an architecture and add a two-dimensional field with the following commands:

```
sim = Simulator();
sim.addElement(NeuralField('field u', [100, 150], 10, -5, 4));
```

The class `NeuralField` is the same as the one used for one-dimensional fields, the dimensionality is determined by the `size` parameter, which is set to `[100, 150]` here. We add two-dimensional Gaussian stimuli (that have the same size as the field and feed into it) so that we can create some localized activation in the field:

```
sim.addElement(GaussStimulus2D('stim u1', [100, 150], 5, 5, 8, 30, 50), ...
  [], [], 'field u');
sim.addElement(GaussStimulus2D('stim u2', [100, 150], 5, 5, 8, 70, 100), ...
  [], [], 'field u');
```

The lateral interactions in the two-dimensional field are realized as a convolution with an interaction kernel over the two-dimensional feature space, analogous to the one-dimensional case. Computing convolutions over two-dimensional fields (or fields of higher dimensionality) is computationally costly, and some care should be taken when designing architectures to avoid excessive computation time. There are two different implementations of the convolution available: The one used in most elements (such as `GaussKernel2D` and `LateralInteractions3D`) is to split up the kernel into one-dimensional components and perform the convolution separately along each dimension. The second implementation is realized in `KernelFFT`, which builds on the fact that a convolution can be described as an element-wise multiplication in Fourier space, and which uses the Fast Fourier Transformation (FFT) implemented in Matlab. The `KernelFFT` element can be used for all the types of convolutions provided in the other elements (Gaussian kernel and difference of Gaussians with or without global inhibition), and for any dimensionality of the kernel.

Which of these implementations is faster depends on the details of the model. The computation cost of the FFT approach is independent of kernel size and type (Gaussian or difference of Gaussians), and depends only on the size of the field. It is inherently performed in a circular fashion; a non-circular convolution can be emulated, but can be very slow. The standard approach is faster for non-circular convolutions with small interaction kernels. Here, we will use the FFT approach. The lateral interactions for `field u` are then specified as follows:

```
sim.addElement(KernelFFT('u -> u', [100, 150], ...
  [5, 5], 20, [10, 10], 20, -0.05), ...
  'field u', 'output', 'field u', 'output');
```

The parameters of the `KernelFFT` constructor specify that it is adjusted for an input of size `[100, 150]`, and give width and amplitude parameters for the Gaussian excitatory component, Gaussian inhibitory component, and global component of the interaction kernel. The width parameters are given as vectors, with one entry for each dimension of the input space. Note that to obtain global inhibition, the amplitude of the global component must be set to a negative value. The following arguments to the outer function `addElement` specify that the kernel receives input from `field u` and projects back to it, using the component `output` for both projections.

Alternatively, we could use an element of class `LateralInteractions2D` to obtain the exact same pattern of lateral interactions:

```
sim.addElement(LateralInteractions2D('u -> u', [100, 150], ...
  5, 5, 20, 10, 10, 20, -0.05), ...
  'field u', 'output', 'field u', 'output');
```

Note that the `LateralInteractions2D` element also provides the sums over the different dimensions of the input as additional components `horizontalSum`, `verticalSum`, and `globalSum`. The summation over the input is needed to compute the global interactions efficiently, but they can also be used for projections to one-dimensional fields, as described below. (Technically, the element would not need to compute both horizontal and vertical sum for the global interactions, but both are provided for symmetry).

If you do not need the full interaction pattern of local excitation, local surround inhibition, and global inhibition in your model, you may use either the `GaussKernel2D` or `MexicanHatKernel2D` elements to implement the lateral interactions.

17.2 Projections between Fields of Different Dimensionality

To describe the typical mechanisms for projections between one- and two-dimensional fields, we first add a one-dimensional field to the architecture. We assume that this field is defined over the same space that forms the second (horizontal) dimension of `field u`. We call this field `field w`:

```
sim.addElement(NeuralField('field w', [1, 150], 10, -5, 4));
```

We further include lateral interactions for this field and a Gaussian stimulus:

```
sim.addElement(...
  LateralInteractions1D('w -> w', [1, 150], 5, 15, 12.5, 15, 0, true), ...
  'field w', 'output', 'field w', 'output');
sim.addElement(GaussStimulus1D('stim w1', [1, 150], 5, 3, 50, true), ...
  [], [], 'field w', 'output');
```

We now want to implement a projection from `field u` to `field w`. Since the vertical dimension of `field u` has no correspondence in `field w`, `field u` is summed over this dimension first, yielding a one-dimensional output. To this end, we add an element of class `SumDimension` as follows:

```
sim.addElement(SumDimension('sum u', 1, [1, 150]), 'field u', 'output');
```

We specify in the parameters that the element should compute the sum over the first (vertical) dimension of its input, and that the result is a vector of size `[1, 150]`. The actual projection to `field w` is now mediated by another Gaussian interaction kernel (reflecting synaptic spread generally found in biological neural systems):

```
sim.addElement(GaussKernel1D('u -> w', [1, 150], 5, 0.5), ...
  'sum u', 'output', 'field w', 'output');
```

The interaction kernel is one-dimensional, with the size parameter matched to `field w`. This is the case because the output of `field u` is already reduced to one dimension by the summing operation before the interaction kernel is applied. The kernel sigma is set to five, the amplitude of the projection to 0.5. Note that the amplitude for such projections from higher- to lower-dimensional fields should typically be chosen rather small, because the summing operation will yield local amplitudes much larger than the output of a one-dimensional field.

The reverse projection is likewise mediated by a Gaussian interaction kernel. In this case, we begin with the convolution, and then expand the result:

```
sim.addElement(GaussKernel1D('w -> u', [1, 150], 5, 5), ...
  'field w', 'output');
sim.addElement(ExpandDimension2D('expand w -> u', 1, [100, 150]), ...
  'w -> u', 'output', 'field u', 'output');
```

We again use a one-dimensional convolution kernel with size `[1, 150]`, which receives input from `field w`. This convolution does initially not project to any other elements, the projection to `field u` is only added in the next step. In this step, an `ExpandDimension2D` element

is added to scale the result of the convolution up to the size of `field u`. The element, labeled `expand w -> u`, receives as input the `output` component of `w -> u`, and expands it along dimension 1 to a size of `[100, 150]`. To do that, it vertically fills a matrix of the specified size with copies of the input vector. For expansion along the second dimension, the `ExpandDimension2D` would also rotate the input appropriately. Note that for more general expansions (with more than two dimensions), there is the generic element `ExpandDimension` with additional parameters.

In the script for this example, we then run the simulator and plot the activations at two points in time. The simulator effectively performs a selection decision between two stimuli in `field u`, which is biased by subthreshold input to `field w` through the bidirectional coupling between the fields.

18 General Hints for Efficient DNF Architectures

18.1 Connecting Fields of Different Dimensionality

When connecting a higher-dimensional and lower-dimensional field, all computationally costly operations – such as convolutions – should be performed in the lower dimensional space whenever possible. This means:

- When projecting from a 2D field to a 1D field, one should first compute the sum over the 2D field's output, and then apply the convolution (or other operation) to this sum.
- When projecting from a 1D field to a 2D field, one should first perform the convolution on the output of the 1D field, and then expand the result of this convolution to two dimensions.

18.2 Scaling and Re-using Operations

Many connective elements have an `amplitude` parameters that scales the output of that element (this is the case for all elements that are expected to be used for direct connections between fields). If you need to have the output of an element without `amplitude` parameter scaled, you can do so by feeding it to an element of class `ScaleInput`.

The `ScaleInput` element may also be employed for re-using other elements in multiple projections: Assume for instance that you have a two-dimensional field that should have an excitatory projection to itself and to one other two-dimensional field, both mediated by Gaussian kernels. If the Gaussian for the two projections should have the same width, it is considerably more efficient to perform the convolution only once, and then scale the result twice for the different projections. The amplitude of the Gaussian kernel itself should then be set to 1.0, so that the amplitudes of the `ScaleInput` elements directly reflect the amplitude of the respective projection.

18.3 Order of Elements

The `step` function of the elements in a `Simulator` object is called in the order that the elements were added to the object. This order may affect the exact behavior of the whole dynamical system (although the numerical differences in each step will be small for reasonable step sizes). We suggest the following standard for element order:

- all external stimuli

- all connective elements between the stimuli and the fields (if connective elements are set up in a chain, the elements of that chain should be added in descending order)
- all dynamic elements (neural fields and memory traces)
- all connective elements between fields (in descending order for chains of connective elements)
