# Supplementary figures and images for: Computational modelling reveals the influence of object similarity and proximity on visually guided movements
Source: PeerJ. 2025 Feb 25;13:e18953. doi: 10.7717/peerj.18953 (PMC11869896; doi:10.7717/peerj.18953)

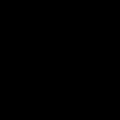

Supplement: Supplemental Information 1 [file peerj-13-18953-s001.zip › CoRLEGO Simulation Code/Input Images/NoTargetImage.png]

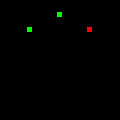

Supplement: Supplemental Information 1 [file peerj-13-18953-s001.zip › CoRLEGO Simulation Code/Input Images/redTargetOnTheRight.png]

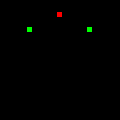

Supplement: Supplemental Information 1 [file peerj-13-18953-s001.zip › CoRLEGO Simulation Code/Input Images/redTargetInTheMiddle.png]

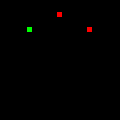

Supplement: Supplemental Information 1 [file peerj-13-18953-s001.zip › CoRLEGO Simulation Code/Input Images/greenTargetOnTheLeft.png]

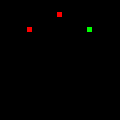

Supplement: Supplemental Information 1 [file peerj-13-18953-s001.zip › CoRLEGO Simulation Code/Input Images/greenTargetOnTheRight.png]

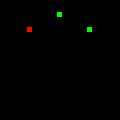

Supplement: Supplemental Information 1 [file peerj-13-18953-s001.zip › CoRLEGO Simulation Code/Input Images/redTargetOnTheLeft.png]

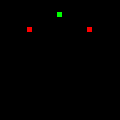

Supplement: Supplemental Information 1 [file peerj-13-18953-s001.zip › CoRLEGO Simulation Code/Input Images/greenTargetInTheMiddle.png]

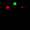

Supplement: Supplemental Information 1 [file peerj-13-18953-s001.zip › CoRLEGO Simulation Code/experiment_images_24/image14.png]

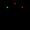

Supplement: Supplemental Information 1 [file peerj-13-18953-s001.zip › CoRLEGO Simulation Code/experiment_images_24/image01.png]

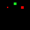

Supplement: Supplemental Information 1 [file peerj-13-18953-s001.zip › CoRLEGO Simulation Code/experiment_images_24/image15.png]

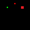

Supplement: Supplemental Information 1 [file peerj-13-18953-s001.zip › CoRLEGO Simulation Code/experiment_images_24/image03.png]

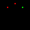

Supplement: Supplemental Information 1 [file peerj-13-18953-s001.zip › CoRLEGO Simulation Code/experiment_images_24/image17.png]

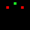

Supplement: Supplemental Information 1 [file peerj-13-18953-s001.zip › CoRLEGO Simulation Code/experiment_images_24/image16.png]

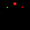

Supplement: Supplemental Information 1 [file peerj-13-18953-s001.zip › CoRLEGO Simulation Code/experiment_images_24/image02.png]

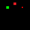

Supplement: Supplemental Information 1 [file peerj-13-18953-s001.zip › CoRLEGO Simulation Code/experiment_images_24/image06.png]

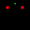

Supplement: Supplemental Information 1 [file peerj-13-18953-s001.zip › CoRLEGO Simulation Code/experiment_images_24/image12.png]

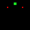

Supplement: Supplemental Information 1 [file peerj-13-18953-s001.zip › CoRLEGO Simulation Code/experiment_images_24/image13.png]

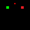

Supplement: Supplemental Information 1 [file peerj-13-18953-s001.zip › CoRLEGO Simulation Code/experiment_images_24/image07.png]

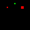

Supplement: Supplemental Information 1 [file peerj-13-18953-s001.zip › CoRLEGO Simulation Code/experiment_images_24/image11.png]

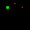

Supplement: Supplemental Information 1 [file peerj-13-18953-s001.zip › CoRLEGO Simulation Code/experiment_images_24/image05.png]

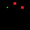

Supplement: Supplemental Information 1 [file peerj-13-18953-s001.zip › CoRLEGO Simulation Code/experiment_images_24/image04.png]

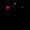

Supplement: Supplemental Information 1 [file peerj-13-18953-s001.zip › CoRLEGO Simulation Code/experiment_images_24/image10.png]

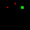

Supplement: Supplemental Information 1 [file peerj-13-18953-s001.zip › CoRLEGO Simulation Code/experiment_images_24/image21.png]

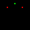

Supplement: Supplemental Information 1 [file peerj-13-18953-s001.zip › CoRLEGO Simulation Code/experiment_images_24/image09.png]

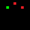

Supplement: Supplemental Information 1 [file peerj-13-18953-s001.zip › CoRLEGO Simulation Code/experiment_images_24/image08.png]

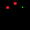

Supplement: Supplemental Information 1 [file peerj-13-18953-s001.zip › CoRLEGO Simulation Code/experiment_images_24/image20.png]

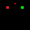

Supplement: Supplemental Information 1 [file peerj-13-18953-s001.zip › CoRLEGO Simulation Code/experiment_images_24/image22.png]

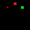

Supplement: Supplemental Information 1 [file peerj-13-18953-s001.zip › CoRLEGO Simulation Code/experiment_images_24/image23.png]

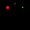

Supplement: Supplemental Information 1 [file peerj-13-18953-s001.zip › CoRLEGO Simulation Code/experiment_images_24/image18.png]

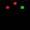

Supplement: Supplemental Information 1 [file peerj-13-18953-s001.zip › CoRLEGO Simulation Code/experiment_images_24/image24.png]

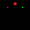

Supplement: Supplemental Information 1 [file peerj-13-18953-s001.zip › CoRLEGO Simulation Code/experiment_images_24/image19.png]

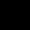

Supplement: Supplemental Information 1 [file peerj-13-18953-s001.zip › CoRLEGO Simulation Code/experiment_images_24/targetblank.png]

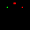

Supplement: Supplemental Information 1 [file peerj-13-18953-s001.zip › CoRLEGO Simulation Code/imagetech/Images/greenTargetonleft_smalltsmalldsmalld.png]

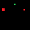

Supplement: Supplemental Information 1 [file peerj-13-18953-s001.zip › CoRLEGO Simulation Code/imagetech/Images/image.png]

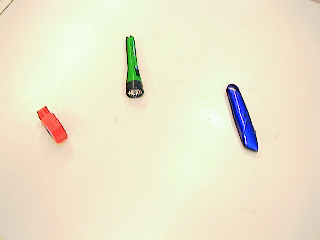

Supplement: Supplemental Information 1 [file peerj-13-18953-s001.zip › CoRLEGO Simulation Code/All in one/examples/sceneSnapshot1.png]

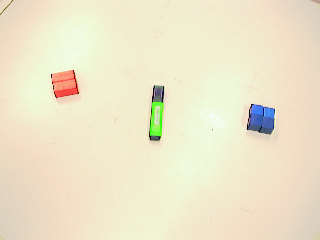

Supplement: Supplemental Information 1 [file peerj-13-18953-s001.zip › CoRLEGO Simulation Code/All in one/examples/sceneSnapshot3.png]
