# Supplementary material for: Computational modelling reveals the influence of object similarity and proximity on visually guided movements
Source: PeerJ. 2025 Feb 25;13:e18953. doi: 10.7717/peerj.18953 (PMC11869896; doi:10.7717/peerj.18953)
Supplement: Supplemental Information 2 — Each data point represents the average performance across trials for a given item combination (dd, Dd, DD) under different size conditions (small: blue squares, large: red stars). The upper panels show IL (interaction latency) in terms of time (in steps), while the lower panels depict MD (movement distance) in terms of distance (in pixels). The solid blue line corresponds to the small target condition, and the dashed red line represents the large target condition. [file peerj-13-18953-s002.pdf]

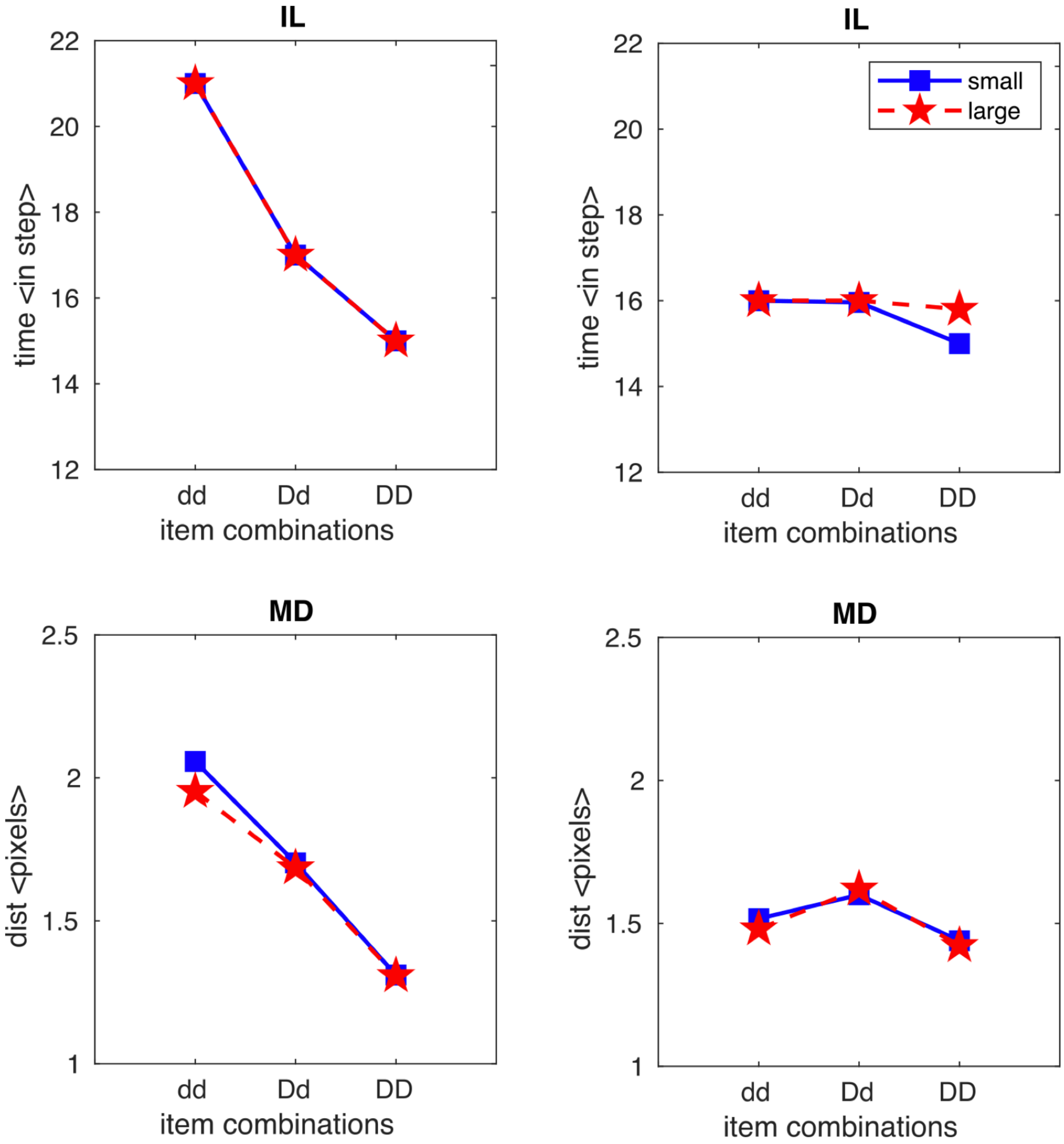


Figure S1. IL (top) and MD (bottom) of the target-in-the-middle trials for the simulation with movement onset scaling factor values as in Fig.6 (left) and Fig.7 (right).
